# Psychometric properties of the Child and Adolescent PsychProfiler v5: a measure for screening 14 of the most common DSM-5 disorders

**DOI:** 10.3389/fpsyg.2024.1267711

**Published:** 2024-08-30

**Authors:** Shane Langsford, Rapson Gomez, Stephen Houghton, Leila Karimi

**Affiliations:** ^1^Psychological & Educational Consultancy Services, Perth, WA, Australia; ^2^School of Health and Biomedical Sciences, RMIT University, Melbourne, VIC, Australia; ^3^School of Health and Biomedical Sciences, Federation University, Melbourne, VIC, Australia; ^4^Graduate School of Education, University of Western Australia, Perth, WA, Australia; ^5^School of Medicine and Healthcare Management, Caucasus University, Tbilisi, Georgia

**Keywords:** Child and Adolescent PsychProfiler, factor structure, psychometric properties, parent ratings, screening, DSM-5

## Abstract

**Introduction:**

The Child and Adolescent PsychProfiler version 5 (CAPP v5, 2014) is a measure for screening 14 common DSM-5 disorders in children and adolescents. The separation of Attention-Deficit/Hyperactivity Disorder (ADHD) and Specific Learning Disorder (SLD) by subtype results in 17 screening scales covering the 14 disorders. Theoretically then, the CAPP v5 should have a 17-factor structure, however, to date no published study has confirmed this. Additionally, there has been no comprehensive evaluation of the reliability and validity of the screening scales in this measure. These were examined across two different studies. Study 1 examined support for the 17-factor model of the parent-report version of the CAPP (CAPP-PRF) in a large group of adolescents from the general community. It also examined the internal consistency reliability and discriminant validity of the factors in this measure. Study 2 examined the validity of these factors in a clinic-referred group of adolescents.

**Methods:**

In Study 1, 951 parents completed the CAPP-PRF on behalf of their adolescents [mean (standard deviation) = 14.54 years (1.66 years)]. In Study 2, 173 parents completed the CAPP-PRF on behalf of their clinic-referred adolescent children [mean (standard deviation) = 14.5 years (1.84 years)]. Adolescents also completed a number of measures and tests for the purpose of assessing their behavior, IQ, and academic abilities.

**Results:**

The results in Study 1 supported a 17-factor model, and virtually all of the factors in this model showed acceptable reliability (alpha and omega coefficients), and discriminant validity. Study 2 demonstrated good support for the validity of the scales in the CAPP-PRF.

**Discussion:**

These findings indicate acceptable psychometric properties for the CAPP-PRF, and its utility for screening the more common DSM-5 disorders in children and adolescents.

## 1 Introduction

The PsychProfiler (Langsford et al., 2007, 2014) is a comprehensive screening instrument for the simultaneous investigation of 20 of the most common disorders found in children, adolescents, and adults. Details of this measure are available at www.psychprofiler.com, and in Supplementary Table S1. The PsychProfiler consists of two conceptually similar instruments: the Child and Adolescent PsychProfiler (CAPP) and the Adult PsychProfiler (APP). The theoretical basis of the CAPP in provided in the *PsychProfiler* Manual (Langsford et al., 2014, pp. 11–15), and for convenience, it is reproduced as part of Supplementary Table S1. In brief, the different versions of the PsychProfiler were developed for the quick screening of the common DSM-5 disorders in children, adolescents, and adults. Consequently, it does not measure a single construct as such, but comprises scales measuring the common disorders listed in the DSM-5. To that extent, it measures several different psychopathology constructs aligned to disorders in the DSM-5.

The current study focuses on the CAPP, which is used for screening children and adolescents aged 2 to 18 years. The CAPP has self-report (SRF), parent-report (PRF), and teacher-report (TRF) versions that screen for 14 of the most common DSM-5 disorders, namely: Attention-Deficit/Hyperactivity Disorder (ADHD), Oppositional Defiant Disorder (ODD), Conduct Disorder (CD), Specific Learning Disorder (SLD), Autism Spectrum Disorder (ASD), Language Disorder (LD), Speech Sound Disorder (SSD), Generalized Anxiety Disorder (GAD), Persistent Depressive Disorder (PDD), Separation Anxiety Disorder (SAD), Obsessive-Compulsive Disorder (OCD), Posttraumatic Stress Disorder (PTSD), Anorexia Nervosa (AN), and Bulimia Nervosa (BN). Separate scales are included for ADHD (Predominantly Inattentive Presentation [ADHDI] and ADHD Predominantly Hyperactive–Impulsive Presentation [ADHDHI]), and for SLD with impairment in Reading, SLD with impairment in Written Expression, and SLD with impairment in Mathematics. Thus, there are 17 screening scales covering the 14 disorders.

In all versions of the CAPP, items in the different screening scales correspond to the DSM 5 disorder symptoms with the same name. Therefore, it is theoretically conceivable that all forms of the CAPP could have a 17-factor structure, with the factors being the 17 screening scales corresponding to their DSM-5 counterparts. A cursory confirmatory factor analytic investigation by Lawrence et al. (2020) link color supported the unidimensionality of the individual screening scales in the CAPP-PRF. However, to date no study has formally explored the factor structure of the CAPP-PRF with all the 17 screening scales together. Confirming the presumed 17-factor structure and identifying a credible structural model would be helpful for its continuing development and for exploration of its other psychometric properties. Given this, the aim of the current study was to examine support for the 17-factor structural model for the CAPP-PRF, and related to this, the reliability (alpha and omega coefficients), and validities of the factors in the model.

## 2 Background information

### 2.1 Existing psychometric properties of the CAPP

All three forms of the CAPP have 126 items, and are rated on a six-point Likert scale (never = 0, rarely = 1, sometimes = 2, regularly = 3, often = 4, and very often = 5). With the exception of one item (item number 39), these items are allocated only to their respective clinical scales. Item number 39 is not assigned to any factor and was included in the instrument for rater-reliability purposes and can therefore be considered a redundant item. When this redundant item is removed, the CAPP comprises 125 items for clinical screening. For all forms of the CAPP there is close alignment between their screening scales and DSM-5 disorder categories with the same names, thereby indicating strong face validity for the different screening scales for the three forms of the CAPP. While there is data available for other psychometric properties of the CAPP-PRF, these are somewhat limited. Given that the focus of the current study was on the CAPP-PRF, summaries of these other psychometric data for the CAPP-PRF will now be reviewed.

The original version of the CAPP was subjected to a rigorous psychometric analysis and has been found to “be reliable and valid” (Langsford et al., 2014; p. 51). Whilst the PsychProfiler Manual includes information supporting inter-rater reliability, clinical calibration, the application for a six-point scale for the ratings of the items, and suitable readability for use by children and adolescents. An unpublished study of the CAPP-PRF, conducted after the printing of the PsychProfiler Manual in 2014, has reported good to adequate support for the unidimensionality for the different screening scales; fair to moderate interrater reliability between parent and self-ratings, and appropriate concurrent and criterion validity for virtually all of the screening scales (Lawrence et al., 2020).

### 2.2 Limitations of existing psychometric properties of the CAPP

Although there is a range of psychometric information available for the CAPP-PRF, a notable omission is that there are no published data on the factor structure of the CAPP-PRF. In the absence of this, it could be argued that the soundness of the existing psychometric properties of the CAPP-PRF may be questionable. A correct understanding of the optimum factor structure of a questionnaire and the application of this structure is necessary for evaluating its other psychometric properties. Thus, in the context of the CAPP-PRF, the relevant question, based on the presence of 17 screening scales, is “Do the items in the CAPP-PRF load on their 17 designated latent factors, as proposed for the CAPP?” Generally, to confirm or establish the factor structure, the independent cluster confirmatory factor analysis (ICM-CFA) is used. Additionally, while there are some data on the validity of CAPP-PRF, this existing information can be seen as limited when viewed against the Standards for Educational and Psychological Testing or “*Standards*,” published by the American Educational Research Association, American Psychological Association, and National Association for Measurement in Education (AERA, APA, NCME, 2014). The purpose of the *Standards* “is to provide criteria for the development and evaluation of tests and testing practices and to provide guidelines for assessing the validity of interpretations of test scores for the intended test uses” (p. 1). As related to test validation, this document focuses on different aspects of validity, and not on distinct types of validity. As summarized by Hawkins et al. (2020), these include evidence based on test content (i.e., the relationship of the item themes, wording and format with the intended construct, including administration process), response processes (the cognitive processes and interpretation of items by respondents and users, as measured against the intended construct), internal structure (the extent to which item interrelationships conform to the intended construct), relations to other variables (the pattern of relationships of test scores to external variables as predicted by the intended construct), and consequences of testing (intended and unintended consequences, as can be traced to a source of invalidity such as construct under-representation or construct-irrelevant variance) as being needed for interpreting and using test scores.

As an example of such an application, we highlight how the Beck Youth Inventories, Second Edition (BYI-2; Beck et al., 2005) for children and adolescents 7 to 18 years of age was developed and validated. The BYI-2 comprises five self-report inventories, measuring depression (Beck’s Depression Inventory for Youths; BDI-Y), anxiety (Beck’s Anxiety Inventory for Youths; BSCI-Y), anger (Beck’s Anger Inventory for Youths; BANI-Y), disruptive behavior (Beck’s Disruptive Behavior Inventory for Youths; BDBI-Y), and self-concept (Beck’s Self-Concept Inventory for Youths; BCSI-Y). The manual for the BYI-2 begins with details of the theoretical background related to this individual questionnaire, including: previous research on the assessment of the disorders in the individual questionnaires; detailed descriptions of the individual measures; and discussion on the clinical application and interpretation of the scores for the individual questionnaires, including their administration, and scoring. It then covers comprehensively how the BYI-2 was developed and standardized, and details of its reliability (internal consistency, standard error of measurement, and test–retest), and validity (evidence based on internal structure; evidence based on relations to other variables, and evidence based on group differences). However, it is to be recognized that in general, validation of a measure is an on-going process.

To some degree, for the CAPP-PRF, the evidence based on test content and response processes have already been established, and are presented in the PsychProfiler Manual (Langsford et al., 2014, p. 51). As mentioned previously, the Manual includes information supporting clinical calibration, the application for a six-point scale for the ratings of the items, and suitable readability for use by children and adolescents. In contrast, there is little data supporting evidence for internal structure, relations to other variables, and consequences of testing. Consequently, they need to be established for reliable interpretation and use of the scores from the CAPP-PRF. The establishment of these are the primary goals of this paper.

### 2.3 Aims of the study

This is a multi-study paper, involving two studies: Study 1 and Study 2. For Study 1,the overall aim was to use ICM-CFA to examine support for the 17-factor structure of the CAPP-PRF for a group of adolescents from the general community. Thus, the focus in Study 1 relates to evidence for internal structure. The factors in the model were ADHDHI, ADHDI, CD, ODD, SLD-R, SLD-W, SDL-M, ASD, LD, SSD, GAD, PDD, SAD, PTSD, OCD, AN, and BN, with each scale corresponding to the symptoms of the DSM-5 disorder with the same name. However, given the complexity of the model tested (125 items, with each item being rated on a six-point scale, and these items loading on 17 factors), we were apprehensive about the ability of the model to show admissible solution. Related to this, we decided that if this was not found, the output of the original 17-factor model would be re-examined and revised for an admissible solution, with adequate global fit. Additionally, the factors in the model had to show clarity (salience and significance of the loadings on their designated factors), reliability (alpha and omega coefficients), and discriminant validity.

For Study 2, we examined the validity of the clinical scales in the CAPP-PRF in a clinic-referred group of adolescents. Consequently, the focus of Study 2 was on the evidence for relations to other variables. As noted by Hawkins et al. (2020), the evidence for this as specified in the *Standards* (AERA, APA, NCME, 2014) include convergent evidence (i.e., relationships between items and scales of the same or similar structure), discriminant evidence (i.e., assessments measuring different constructs determined to be sufficiently uncorrelated), criterion-referenced evidence (i.e., how accurately scores predict criterion performance) and evidence for group differences (i.e., relationships of scores with background characteristics). In Study 2, parents completed the Conners-3-P, and adolescents completed the Beck Youth Inventories, Second Edition, the Wechsler Intelligence Scale for Children, Fifth Edition (Wechsler, 2016a) and the Wechsler Individual Achievement Test, Third Edition (Wechsler, 2016b). Thus, the study had the capacity to examine evidence for convergent, discriminant, and criterion-referenced validity.

Together, therefore, the study examined the factor structure (internal validity) of the CAPP-PRF, and the reliability (alpha and omega coefficients), and discriminant and criterion validities of the factors in our selected CAPP-PRF model. We expected that in addition to the support of a 17-factor model, the factors in the model would show adequate clarity (items loading significantly and saliently on their respective designated factors), internal consistency, and validity (discriminant and criterion).

## 3 Method

### 3.1 Participants

The participants in Study 1 comprised of 951 parents of adolescents who completed the CAPP-PRF either online or as a paper questionnaire. The mean age (*SD*, range) of participants was 14.54 years (1.66 years; 12.01 years to 17.99 years). There were 572 (60.1%) boys, (mean age = 14.54 years, *SD* = 1.70 years) and 372 (39.1%) girls (mean age = 14.52 years, *SD* = 1.60 years), and no gender information for 7 (0.7%) of adolescents. There was no significant difference for age across boys and girls*, t*(*df* = 942) = 0.188, *ns*.

The participants in Study 2 were 173 clinic-referred adolescents (age range from 12 years to 17 years; mean age 14.57 years, *SD* = 1.84 years), of which 112 (64.7%) were boys, (mean age = 14.40 years, *SD* = 2.30 years) and 61 (35.3%) were girls (mean age = 14.69 years, *SD* = 1.80 years). There was no significant difference for age across these groups*, t*(*df* = 171) = 0.842, *ns*. For this group, the parents completed a paper CAPP-PRF as part of the overall assessment requested at the clinic. Additional measures covering the adolescents’ behavior, IQ, and academic abilities were also completed as appropriate by parents and adolescents (details are provided in the procedure section).

For both groups, no selection criteria restricted participation.

### 3.2 Measures

For Study 1, parents completed only the CAPP-PRF (Langsford et al., 2014). For Study 2, parents completed the CAPP-PRF and Conners 3-P (Conners, 2008); the adolescents who were rated by their parents completed the Beck Youth Inventories, Second Edition (BYI-2; Beck et al., 2005) and were also administered the Wechsler Intelligence Scale for Children-Fifth Edition (WISC-V; Wechsler, 2016a), and the Wechsler Individual Achievement Test, Third Edition (WIAT-III; Wechsler, 2016b).

#### 3.2.1 Parent report form Child and Adolescent PsychProfiler (CAPP-PRF)

The PRF CAPP was described in detail in the introduction. Given data availability, this study used the scores on this measure for adolescents aged 12–17 years (Langsford et al., 2014).

#### 3.2.2 The Beck Youth Inventories, second edition (BYI-2)

As mentioned previously, the Beck Youth Inventories, Second Edition (BYI-2; Beck et al., 2005) is for children and adolescents 7 to 18 years of age. It comprises five self-report inventories, measuring depression (Beck’s Depression Inventory for Youths; BDI-Y), anxiety (Beck’s Anxiety Inventory for Youths; BAI-Y), anger (Beck’s Anger Inventory for Youths; BANI-Y), disruptive behavior (Beck’s Disruptive Behavior Inventory for Youths; BDBI-Y), and self-concept (Beck’s Self-Concept Inventory for Youths; BCSI-Y). Each inventory has 20 items, resulting in 100 items in total. Individuals rate all 100 items in terms of the extent to which each statement describes them on a 4-point Likert scale (i.e., “0 = never,” “1 = sometimes,” “2 = often,” “3 = very often”). All five inventories have good construct validity, high reliability (coefficient alpha ranging from 0.86 to 0.96), and high test–retest reliability (coefficients ranging from 0.74 to.93; Beck et al., 2005), thereby supporting their psychometric properties (factor structure, reliability, and validity), and use.

The BDI-Y includes items covering negative thoughts, feelings of guilt and sadness, and sleep issues (e.g., “I have trouble sleeping”); the BAI-Y includes items covering concerns and apprehension regarding school, the future, reactions from others, losing control, and physiological anxiety symptoms (e.g., “My hands shake”). Thus, the scores for the BDI-Y and the BAI-Y can be considered suitable for evaluation of the criterion validity of CAPP-PRF PDD and GAD screening scales, respectively. The BANI-Y focuses on feelings of hatred and anger as well as thoughts of unjust or unfair treatment (e.g., “I get mad and stay mad”); and the BDBI-Y includes items relating to behaviors and attitude associated with ODD and CD (e.g., “I hurt people”). Thus, both the BDBI-Y and to a lesser degree, the BANI-Y can be considered suitable for evaluation CAPP-PRF ODD and CD. The BSCI-Y focuses on self-perception, such as competence, potency, and positive self-worth (e.g., “I like my body”). For each inventory, item scores are summed and converted to *T*-Scores, with higher scores indicating greater severity. The scores for all five inventories have shown adequate convergent validity with scales measuring related constructs (Beck et al., 2005), and ability to distinguish between clinical and nonclinical samples (Thastum et al., 2009).

For all measures, BDI-Y, BAI-Y, BANI-Y, BDBI-Y, and BSCI-Y, there is evidence that their scores are able to distinguish individuals (children and adolescents) with and without the relevant disorder that they measures (Beck et al., 2005).

#### 3.2.3 The Conners 3-P

The Conners 3–Parent (Conners 3-P; Conners, 2008) is a screening measure used in the diagnosis of ADHD and disorders commonly comorbid with ADHD in children and adolescents aged 6–18 years. While it has scores for a number of correlates associated with ADHD (e.g., learning problems/executive functioning), it also includes four DSM-5 Symptom Scales. These are the DSM-5 ADHD Predominantly Inattentive Presentation Symptom Scale, DSM-5 ADHD Predominantly Hyperactive–Impulsive Presentation Symptom Scale, DSM-5 Conduct Disorder Symptom Scale, and the DSM-5 Oppositional Defiant Disorder Symptom Scale (Conners, 2008). The scores for these scales are highly correlated with the DSM-5 disorders with the same name (Conners, 2008), meaning that they are appropriate for evaluating the convergent and discriminant validity of the CAPP screening scales for ADHDI, ADHDHI, CD, and ODD as they correspond appropriately with DSM-5 symptoms. Indeed, there is evidence that the ADHD related scales are able to distinguish individuals (children and adolescents) with and without the relevant ADHD disorder types (Conners, 2008).

#### 3.2.4 Wechsler intelligence scale for children–fifth edition: Australian and New Zealand standardized edition

The WISC-V is an individually administered test of intelligence for children from 6 years 0 months to 16 years 11 months (Wechsler, 2016a). It provides index scores for cognitive area related to Verbal Comprehension (VCI), Visual Spatial (VSI), Fluid Reasoning (FRI), Working Memory (WMI), and Processing Speed (PSI); and a composite score that represents general intellectual ability (i.e., Full Scale IQ or FSIQ). Existing data indicate that VCI, WMI, and PSI are associated negatively with SLD difficulties in reading (SLDR; Cornoldi et al., 2019), and mathematics (SLDM; Mayes and Calhoun, 2007; Geary, 2011). In addition, FSIQ is associated negatively with SLDM (Rainford et al., 2016). There is also data linking language disorder (LD) with a lower VCI (Cornoldi et al., 2019) and a higher nonverbal IQ (Rice, 2016). Findings such as these suggest that the VCI, WMI, PSI and FSIQ are appropriate for evaluating the criterion validity of the CAPP-PRF screening scales for LD, and to a lesser degree, SLDR, SLDW, and SLDM.

#### 3.2.5 Wechsler individual achievement test, third edition: Australian and New Zealand standardized edition (WIAT-III)

The WIAT-III is an individually administered clinical instrument designed to measure the academic achievement of students who are in kindergarten through to the final year of secondary school (i.e., Year 12), or ages 4 years 0 months to 50 years 11 months. It consists of a total of 16 subtests grouped into listening, speaking, reading, writing, spelling, and mathematical skills. The Australian and New Zealand version of the test was standardized on a sample of 1,360 Australian and New Zealand students and features comprehensive normative information. We used the scores for reading, written expression, and mathematics to examine the criterion validity of the CAPP-PRF screening scales for LD, SLDR, SLDW, and SLDM. Low scores for reading, written expression, and mathematics are generally used for identifying children with reading, written, and mathematic disorders, respectively (Wechsler, 2016b).

### 3.3 Procedure

The PsychProfiler measures (including the CAPP) have a designated website^1^ that can be used by those interested in the online screening of DSM-5 disorders using any of the PsychProfiler forms. The primary users are psychologists, psychiatrists, pediatricians, and the general public. The participants involved in Study 1 provided data through the website. On completion of the PsychProfiler, individuals were requested, if they so wished, to click a statement consenting to their data being used for future research and instrument validation purposes. Only adolescents with CAPP-PRF ratings and consent were included in Sample 1.

Study 2 included adolescent participants seen in a clinic setting in Perth, Western Australia. They were attending the clinic to complete an ADHD, SLD, and/or ASD assessment. These adolescents were referred to the clinic from a variety of sources (e.g., privately by their parents, through their school, or from a general practitioner, pediatrician, or child and adolescent psychiatrist). Parents of the adolescent participants in this sample completed the CAPP-PRF and the Conners3-P as part of the assessment that their child was undergoing, and the adolescent completed the self-report BYI-2. Parents were also provided a Consent Form for signing should they consent to their child’s de-identified data being used for future research and instrument validation purposes. Only information of adolescents whose parents had signed the Consent Form were included in Sample 2. Thus, informed consent was obtained from the parents of all adolescent participants involved in the study.

### 3.4 Statistical analysis

As described earlier, participants in Study 1 and Study 2 constituted different groups. Sample 1 comprised 951 parent ratings of adolescents on the CAPP-PRF. The primary goal of the analysis for this sample was to use Confirmatory Factor Analysis (CFA) to examine the fit of the 17-factor CAPP model. Initially, we used the descriptive module in Jeffreys’ Amazing Statistics Program (JASP Team, 2023) version 0.16.6.0 statistical software to compute the mean and standard deviation scores, and the dispersion statistics of the 125 items of the CAPP. Brown (2015) has suggested that data can be considered to have normal univariate distribution if skewness is between −3 to +3 and kurtosis is between −10 to +10. Streiner and Norman (1995) assert nonnormality can be seen as problematic if ≥80% of responses are at one end of the scale.

M*plus* Version 7 (Muthén and Muthén, 2012) was used to analyze the 17-factor CAPP-PRF model. As each item was scored on a six-point scale, we applied maximum likelihood (ML) extraction. Robust ML was not used as the data was considered not to have non-normality problems (details presented below). At the statistical level, the global fit of this model was examined using the chi-square test. However, as the chi-square statistic is inflated by large sample sizes, several approximate fit indices have been proposed. Among others, commonly used fit indices have included the relative chi square (relative χ^2^) (sometime called normed χ^2^) which is a ratio of the chi-square statistic to the respective degrees of freedom (χ^2^/df), Root Mean Squared Error of Approximation (RMSEA), Comparative Fit Index (CFI), Tucker Lewis Index (TLI), and the Standardized Root Mean Residual (SRMR).

Kline (2005) suggested that for model fit, chi-square, RMSEA, CFI, and SRMR be examined in combination. Although RMSEA, CFI and TLI are frequently used to evaluate model fit, we did not use these in the current study because as these values are derived from the chi-square value, they would also be compromised (Shi et al., 2019). In contrast, SRMR is not derived from the chi-square value (Pavlov et al., 2021). Taking all of this into consideration, the current study used relative χ^2^ and SRMR in combination to evaluate model fit. For relative χ^2^, acceptable values have ranged from less than 2 (Ullman and Bentler, 2012) to less than 5 (Schumacker and Lomax, 2004) to be deemed acceptable. Hu and Bentler (1999) have proposed that for SRMR, values ≥0.80 = acceptable. Despite not relying on the CFI, TLI and RMSEA values for evaluating model fit, we report these values for those interested. For these indices, Hu and Bentler (1999) have proposed that for RMSEA, values <0.06 = good fit, <0.08 = acceptable fit, and > 0.08 to 0.10 = marginal fit. For CFI and TLI, values ≥0.95 = good fit, and ≥ 0.90 = acceptable fit.

Additionally, for model acceptance in this study, it was necessary for the loadings of the indicators in the model to be significant and salient (>0.30; Field, 2013), and for the factors to demonstrate acceptable discriminant validity (*r* < 0.85; Brown, 2015), and acceptable reliability omega coefficients (Zinbarg et al., 2005). Although there are no universally accepted guidelines at present for interpreting omega coefficients, Watkins (2017) proposed that omega values should meet the same standards as alpha coefficients. For alpha coefficients, guidelines for acceptability have ranged from 0.70 and above (Kline, 1998). For the current study we used omega values of at least 0.70 as acceptable. Also, for those interested, we report the alpha reliability coefficients of the 17 factors. Finally, as mentioned earlier, given model complexity, we were apprehensive about the ability of the 17-factor CAPP model to show admissible solution. Related to this, we decided that if this occurred, the model would be revised to achieve a model that had acceptable admissible solution.

Study 2 comprised 171 parent ratings of clinic-referred adolescents on the CAPP-PRF. For this sample, the data used were the total scores for the 17 scales in the CAPP-PRF (Langsford et al., 2014). The primary analysis for this sample examined the validity of these 17 scales. For this, we used SPSS. We examined Pearson’s correlations of the factors in this model with the total scores in the BDI-Y, BAI-Y, BANI-Y, BDBI-Y and BSCI-Y (BYI-2; Beck et al., 2005); DSM-5 symptom scales (DSM-5 ADHD Predominantly Inattentive Presentation Symptom Scale, DSM-5 ADHD Predominantly Hyperactive–Impulsive Presentation Symptom Scale, DSM-5 Conduct Disorder Symptom Scale, and the DSM-5 Oppositional Defiant Disorder Symptom Scale) of the parent version of Conners-3 (Conners, 2008); and the WISC-V (Wechsler, 2016a) composite scores for VCI, VSI, FRI, WMI, PSI, and, FSIQ. Correlations were also computed for the factors in the CAPP-PRF model with the diagnosis of specific learning disorders for reading, written expression, and mathematics. These correlations values were squared to represent the proportional relations between variables as they are empirically sound for inferring magnitude of effect (Baguley, 2009).

## 4 Results

### 4.1 Study 1

#### 4.1.1 Sample size requirements

Soper’s (2022) software for computing sample size requirements for the CFA models was used to evaluate the sample size required for the sample in Study 1. The anticipated effect size was set at 0.3, power at 0.8, the number of latent variables at 17, the number of observed variables at 123, and probability at 0.05. The analysis recommended a minimum sample size of 462. Therefore, with *N* = 951 our sample size was more than adequate for the current study.

#### 4.1.2 General comments relating to the tables in this study

Considering that the CFA involved 125 items and 17 factors, the summaries of our tables are lengthy. In view of this, all tables for the study are presented as Supplementary materials.

#### 4.1.3 Descriptive and dispersion statistics of the CAPP items

Supplementary Table S2 shows the mean and standard deviation (SD) scores, and the dispersion statistics of the 125 items of the CAPP. The overall mean (*SD*) score for the 125 items was 1.612 (0.761). Additionally, the median and mode scores across the 125 items were both 1.640. As all items were rated on a six-point Likert scale (never = 0, rarely = 1, sometimes = 2, regularly = 3, often = 4, and very often = 5), the overall, mean, medium and mode scores suggest that in general, individuals were endorsing either rarely or sometimes as their responses. Overall, therefore, the participants in the study had relatively low pathology.

The skewness scores ranged from −0.64 to 5.22, with only 3 items having values outside −3 to +3. For kurtosis, the scores ranged from −1.42 to 32.65, with only five items having values outside −10 to +10. As suggested, data can be considered to have normal univariate distribution if skewness is between −3 to +3 and kurtosis is between −10 to +10 (Brown, 2015), and that nonnormality can be considered problematic if ≥80% of responses are at one end of the scale (Streiner and Norman, 1995). In light of this, our skewness and kurtosis findings can be interpreted as reflecting relatively normal distribution. This, in part, explains why we used ML and not robust ML in the CFA. In terms of missing values for the items, out of 951 possible item responses, they ranged from 3 (for item #3) to 89 (for item #124). We used full-information maximum likelihood (FIML; e.g., Graham, 2009) to handle missing values.

#### 4.1.4 Fit indices of the 17-factor CAPP model

The fit values for the 17-factor CAPP model were: ML*χ*^2^ (*df* = 7,244) = 29527.720; relative *χ*^2^ = 0.4.034; SRMR = 0.83; CFI = 0.740; TLI = 0.731; RMSEA = 0.057, 90% CI [0.056, 0.058]. Based on current guidelines, the relative *χ*^2^ and the SRMR values (used here for evaluating global model fit) can be interpreted as indicating acceptable model fit. Although not used here for evaluating model fit, it should be noted that the RMSEA value (0.057) also indicated good fit. Despite these positive findings, our output indicated that our model was inadmissible as the correlations between GAD and PDD, and AN with BN were above 1, i.e., 1.034 and 1.050, respectively. This problem was resolved by fixing the correlations between these factors to 1 (with the variance for all these factors also set at 1). This is appropriate as reports of high associations between these pairs of correlations are generally reported (e.g., Achenbach and Rescorla, 2001; Fairburn and Bohn, 2005). The revised model produced an admissible solution: Relative χ^2^ and SRMR values were 4.075 and 0.082, respectively. Thus, our revised 17-factor model showed acceptable fit and the RMSEA value also indicated good fit at 0.057.

#### 4.1.5 Pattern of factor loadings in the revised 17-factor PP model

Supplementary Table S3 shows the pattern of factor loadings in the revised 17-factor CAPP model. As shown in the table, all items loaded significantly and saliently (≥ 0.30) on their respective designated factors. Thus, the factors in the model were clearly defined.

#### 4.1.6 Correlations of the factors in the revised 17-factor PP model

Supplementary Table S4 shows the correlations of the factors in the revised 17-factor CAPP-PRF model. It shows that, apart from the correlations of SLDR and SLDW with AN, BN and OCD, and SLDW with PTSD, all other correlations were significant. However other than the latent factor correlations that were constrained to 1 (GAD with PDD, and AN with BN), only SLDR and SLDW had correlations >0.85. These findings can be taken to indicate that generally there was good support for discriminant validity across the latent factors, except between GAD with PDD, AN with BN, and SLDR with SLDW; all of which are known to commonly co-occur together.

For other correlations, GAD was associated with all other screening scales, with these associations being of high effect sizes for SAD, ASD, PDD, ODD, AN, BN, OCD, PTSD. SAD was related to all other scales, with the associations being of high effect sizes for GAD, OCD and PTSD scales. ADHDHI was associated with all other scales, with these associations being of high effect sizes for ADHDI, ASD, CD and ODD scales. ADHDI was associated with all other scales, with these associations being of high effect sizes for ADHDHI, ASD, LD, ODD and SLDW scales. ASD was associated with all other screening scales, with these associations being of high effect sizes for GAD, ADHDHI, ADHDI, LD, SSD, PDD, ODD, and PTSD scales. LD was associated with all other screening scales, with these associations being of high effect sizes for ADHDI, ASD, SSD, and all the SLDs (SLDR, SLDW and SLDM) scales. SSD was associated with all other screening scales, with these associations being of high effect sizes for ASD, LD, SLDR and SLDW scales. PDD was associated with all other screening scales, with these associations being of high effect sizes for GAD, ASD, ODD, AN, BN, OCD and PTSD scales. CD was associated with all other screening scales, with these associations being of high effect sizes for ADHDHI and ODD scales. ODD was associated with all other screening scales, with these associations being of high effect sizes for GAD, ADHDHI, ADHDI, ASD, PDD and CD scales. With the exception of ADHDI, SLDR and SLDW, AN was associated with all other screening scales, with these associations being of high effect sizes for GAD, PDD and PTSD scales. With the exception of SLDR and SLDW, BN was associated with all other screening scales, with these associations being of high effect sizes for GAD, PDD AN and PTSD scales. With the exception of SLDR and SLDW, OCD was associated with all other screening scales, with these associations being of high effect sizes for GAD, SAD, PDD and PTSD scales. With the exception of AN, BN and OCD, SLDR was associated with all other screening scales, with these associations being of high effect sizes for LD, SSD, SLDW and SLDM. With the exception of AN, BN, OCD, SLDW was associated with all other screening scales, with these associations being of high effect sizes huge for ADHDI, LD, SSD, SLDR, and SLSM. SLDM was associated with all other screening scales, with these associations being of high effect sizes for LD, SLDR and SLDM scales. PTSD was associated with all other screening scales, with these associations being of high effect sizes for GAD, SAD, ASD, PDD, AN, BN, and OCD scales.

#### 4.1.7 Internal consistency reliability coefficients for the 17 factors in CAPP model

Supplementary Table S4 also includes the factor-based internal consistency reliability omega coefficients of the 17 latent factors. As shown, they ranged 0.809 to 0.927. The internal consistency reliability alpha coefficients ranged from 0.76 to 0.93. These values indicated acceptable internal consistency reliability for all 17 CAPP-PRF factors.

### 4.2 Study 2

#### 4.2.1 Validity of the CAPP scales

Supplementary Table S5 shows the Pearson’s correlation coefficients of the total scores of the 17 CAPP-PRF scales with the total scores for Conners 3-P DSM-5 Symptom Scales. As shown, the CAPP-PRF scores for ADHDI, ADHDHI, CD, and ODD were correlated significantly and positively with the Conners 3-P DSM-5 Symptom Scale scores with the same name. The effect sizes (*r*^2^ or proportional relations between variables) between corresponding ADHDI and ADHDHI scales were relatively larger than the other *r*^2^ values involving other CAPP-PRF scales. These findings support the convergent and discriminant validity of the CAPP-PRF scales for ADHDI and ADHDHI.

Supplementary Table S6 shows the Pearson’s correlation coefficients of the total scores of the 17 CAPP-PRF scales with the total scores for the BYI-2 Scales. As shown, the CAPP-PRF score for GAD was correlated significantly and positively with total BAI-Y anxiety score. The CAPP-PRF score for PDD was also correlated significantly and positively with the total BDI-Y score for depression. The *r*^2^ for these relations were relatively larger than the other *r*^2^ values, involving other CAPP-PRF scales. This indicates supporting the convergent and discriminant validity of the CAPP-PRF GAD and PDD scales.

Supplementary Table S7 shows the Pearson’s correlation coefficients of the total scores of the 17 CAPP-PRF scales with the WISC-V Composite Scores. As shown, the CAPP-PRF LD and SLDR scores were correlated significantly and positively with WISC-V VCI; the CAPP-PRF SLDW correlated significantly and positively with WISC-V WMI and PSI; and the CAPP-PRF SLDM correlated significantly and positively with WISC-V WMI, PSI and FSIQ. The *r*^2^ for these relations were relatively larger than the other *r*^2^ values, involving other CAPP-PRF scales. Therefore, there was support for the criterion validity of the CAPP-PRF LD, SLDR, SLDW, and SLDM scales.

Supplementary Table S8 shows the Pearson’s correlation coefficients of the total scores of the 17 CAPP-PRF scales with the WIAT-III Composite Scores. As shown, the CAPP SLDR correlated significantly and positively with WIAT-III reading deficit; the CAPP SLDW correlated significantly and positively with WIAT-III reading and written expression deficits, and the CAPP SLDM correlated significantly and positively with WIAT-III mathematics deficit. The *r*^2^ for these relations were relatively larger than the other *r*^2^ values, involving other CAPP-PRF scales. Therefore, there was support for the criterion validity of the CAPP SLDR and SLDM scales.

It is also noteworthy that many of the other CAPP scales correlated as theoretically expected with the different external correlates (for example, CAPP AN, BN and PTSD were correlated positively with anxiety and depression). However, the CAPP SSD and OCD scales showed no association with any of the external correlates. Taken together these findings indicate reasonable support for the validity of the CAPP-PRF scales, especially ADHDI, ADHDHI, GAD, PDD, SLDR, SLDW, SLDM, and to a lesser degree LD, AN, BN, and PTSD.

## 5 Discussion

The CAPP has subscales for screening 17 common DSM-5 disorders in children and adolescents and therefore might be considered to have a 17-factor structure. As this structure, or any other structure, has not been fully tested or confirmed for the CAPP, the primary aim of Study 1 was to use CFA to examine the level of support for the 17-factor structure. This was examined for the parent version of the CAPP (CAPP-PRF) in a group of adolescents. It also examined the reliability (alpha and omega coefficients), and discriminant validity of the factors in this model. Study 2 examined support for validity of the CAPP-PRF. In Study 1, from an initial group of parent ratings for 951 adolescents, the findings supported a slightly modified 17-factor model. For this model, all items loaded significantly and saliently on their respectively, designated factors (i.e., the factors in the model were clearly defined). In addition, virtually all of the factors showed good discriminant validity (except between SLDR and SLDW with AN, BN and OCD, and SLDW with PTSD) and good internal consistency reliability (i.e., omega coefficients ranged from 809 to 0.927 and their alpha coefficients ranged from 0.759 to 0.928). In Study 2, most of the factors/scales in the CAPP-PRF also showed evidence supporting their criterion, concurrent and discriminant validity. This was especially so for the CAPP screening scales for ADHDI, ADHDHI, ODD, CD, GAD, PDD, LD, SLDR, SLDW, and SLDM. Overall, our findings extend the existing psychometric properties of the CAPP-PRF that have shown validity evidence related to test content and response and support for clinical calibration and the application for a six-point scale for the ratings of the items, and suitable readability for use by children and adolescents (Langsford et al., 2014; p. 51). Although these do not cover all the psychometric properties, as specified in the *Standards* (AERA, APA, NCME, 2014), taking all these currently known psychometric properties into consideration, it can be argued that the CAPP-PRF is a promising measure for screening the 17 DSM-5 disorders in it, and for evaluating other substantive issues in adolescent psychopathology. Relatedly, we will discuss the practical and clinical implications in relation to the CAPP-PRF’s practical utilization, comorbidity of common child and adolescent disorders, and the Hierarchical Taxonomy of Psychopathology model (HiTOP; Kotov et al., 2017; Ruggero et al., 2019).

### 5.1 Practical implications

Based on all the known psychometric properties of the CAPP-PRF, it can be argued that the CAPP-PRF has satisfactory psychometric properties for use with adolescents. Importantly, as the 17 different screening scales in the CAPP correspond to the symptoms proposed for the DSM-5 disorders with the same name, it could be assumed that for adolescents, the CAPP-PRF screening scales are suitable for adolescent screening of the DSM-5 clinical disorders with the same name. Notwithstanding this, there are limitations to keep in mind when using some screening scales in this measure.

First, there was no support for the discriminant validity between SLDR and SLDW. However, this is not to be unexpected given that specific learning disorder is now presented in the latest DSM as a single disorder rather than segmented by academic achievement area, and high rates of comorbidity between reading and written expression difficulties have long been established (Ehri, 2000; Pagliarini et al., 2015).

Second, although our findings provide support for the criterion validity of the CAPP-PRF ADHDI, ADHDHI, CD, ODD, GAD, PDD, SLDR, SLDW and SLDM, and to a lesser extent for LD, PTSD, AN and BN, we were unable to demonstrate criterion validity for ASD, SSD, SAD, and OCD as these disorders were not contained within the external measures used in Study 2. Nonetheless, these scales did demonstrate theoretically expected associations with the different external correlates (e.g., CAPP AN, BN and PTSD were correlated positively with anxiety and depression). Therefore, a degree of caution is warranted when using the SSD and OCD scales, and to a lesser degree ASD, SAD, PTSD, AN, and BN. Additionally, important other psychometric properties are still missing for the clinical use of the CAPP, including for example information on its predictive validity.

Third, in the CAPP-PRF the different screening scales are measured using items corresponding to their symptoms (including wording in most instances), as presented in DSM-5. All of the items are rated on a six-point Likert scale (never = 0, rarely = 2, sometimes =2, regularly = 3, often = 4, and very often = 5). For calculating screening scores, the item scores were recoded as follows: never, rarely, sometimes = 0; and regularly, often, and very often = 1. Although, there is some exception to these scoring rules (e.g., fighting with a weapon and stealing were considered to be of sufficient severity that “sometimes” was scored as 1), the total scores of items within each CAPP scale produces a screening score for that disorder. When the screening score for a scale exceeds the screening cut-off score, the disorder that it corresponds to is considered to be present. Currently, the cut-off scores for each screening scale are identical with the symptom threshold cut-off scores for them in the DSM-5 (e.g., if the CAPP ODD scale has a score of 4 or more for ODD, then ODD is considered to have a positive screen, as the symptom threshold cut-off score for ODD in the DSM-5 is 4). Given the close alignment of the items in the CAPP screening scales with the symptoms in the corresponding DSM-5 disorders, this practice seems intuitively prudent. However, establishing cut-off scores for clinical disorders in screening scales is more complex. Best practice standards require application of empirically derived diagnostic utility statistics (such as, sensitivity, specificity, positive predictive power and negative predictive power). It is important therefore that clinicians keep this in mind when using the CAPP. Notwithstanding this, our findings do support the use of the CAPP-PRF for screening the common DSM-5 disorders in adolescents.

### 5.2 Implications for comorbidity

With the exception of the screening scales for the eating disorders (AN and BN) and specific learning disorders (SLDR, SLDW, and SLDM), all of the other screening scales were associated with each other and with AN, BN, SLDR, SLDW, and SLDM. Findings also indicated relative high associations for the CAPP scales for GAD with SAD, ASD, PDD, ODD, AN, BN, OCD, PTSD; SAD with GAD, OCD and PTSD scales; ADHDHI with ADHDI, ASD, CD and ODD scales; ADHDI with ADHDHI, ASD, LD, ODD and SLDW scales; ASD with GAD, ADHDHI, ADHDI, LD, SSD, PDD, ODD, and PTSD scales; LD with ADHDI, ASD, SSD, and all the SLDs (SLDR, SLDW and SLDM) scales; SSD with ASD, LD, SLDR and SLDW scales; PDD with GAD, ASD, ODD, AN, BN, OCD and PTSD scales; CD with ADHDHI and ODD scales; ODD with GAD, ADHDHI, ADHDI, ASD, PDD and CD scales; AN with GAD, PDD and PTSD scales; BN with GAD, PDD AN and PTSD scales; OCD with GAD, SAD, PDD and PTSD scales; SLDR with LD, SSD, SLDW and SLDM; SLDW with ADHDI, LD, SSD, SLDR, and SLSM; SLDM with LD, SLDR and SLDM scales; and PTSD with GAD, SAD, ASD, PDD, AN, BN, and OCD scales.

Taken together, the findings presented raise the possibility of a high degree of comorbidity among the disorders in the CAPP (and by extension psychological disorders), with stronger comorbidity among the screening scales that showed relatively high associations. In general, comorbidity was stronger among: (1) internalizing disorders (GAD, PDD, SAD, OCD, and PTSD); (2) eating disorders (AN and BN); (3) between the internalizing disorders and eating disorders, (4) between the externalizing disorders (ADHDHI, ADHDI, CD, and ODD); (5) between the neurodevelopmental disorders (ASD, LD, SSD, SLDR, SLDW. and SLDM); and (6) between the externalizing disorders and most of the neurodevelopmental disorders. Based on these associations, it is conceivable that the CAPP screening scales can be grouped into internalizing (that includes eating disorders), and externalizing (that includes neurodevelopment disorders). To some extent, our findings are consistent with predictions based on the Hierarchical Taxonomy of Psychopathology model (HiTOP; Kotov et al., 2017; Ruggero et al., 2019).

### 5.3 Implications for the HiTOP model

The HiTOP model is a dimensional model of psychopathology, moving upwards from narrow to broader constructs of psychopathology (Kotov et al., 2017; Ruggero et al., 2019). The problems/syndromes/disorders in HiTOP are at five different hierarchical levels. Of relevance to the current study, at the very top is the superspectra or general p*-*factor (Kotov et al., 2017). Just below this are different spectra, followed by the subfactors. The spectra (6 in all) are somatoform, internalizing, thought disorder, disinhibited externalizing, antagonistic externalizing, and detachment. The subfactors for the internalizing spectra are *distress* (e.g., depression, anxiety, and PTSD), *mania* (e.g., bipolar I disorder, and bipolar II disorder), *fear* (e.g., panic disorder, social phobia, and specific phobia, obsessive-compulsive disorder, separation anxiety disorder), *eating pathology* (e.g., anorexia nervosa and bulimia nervosa) and *sexual problems* (e.g., hyperactive sexual desire disorder, and delayed ejaculation). The subfactors for the externalizing spectra are substance abuse (including ADHD) and antisocial behavior (including ODD and CD).

Although the initially proposed HiTOP model (Kotov et al., 2017) did not include a spectrum for neurodevelopmental problems, a recent study supported its inclusion (Michelini et al., 2019). As is evident, these different groups of spectra/subfactors correspond to how the different screening scales of the CAPP have been grouped (based on the magnitude of their correlations) in this current study. In addition, our findings extend the HiTOP model in one important way. They support a neurodevelopmental spectrum (Michelini et al., 2019), that includes ASD, ADHDHI, ADHDI, LD, SSD, SLDR, SLDW, and SLDM. Consequently, the CAPP may potentially be a useful tool for research involving the HiTOP model (Simms et al., 2021).

### 5.4 Limitations

There are limitations in the current study that need to be considered when viewing the findings. The current study examined only the parent reports of the CAPP in a group of adolescents. This measure has been developed for use with children and adolescents between 2 and 18 years of age. Also, the CAPP has screening forms for self-report and teacher-report. Thus, our findings cannot be generalized to parent reports of CAPP in children, or to the self-report and teacher-report forms. Furthermore, the study did not include a wider range of external variables that would have allowed for the evaluation of the criterion validity of all the CAPP screening scales. Other noteworthy limitations include the fact that the findings are based on a single study and the sample of Study 2 was from the same clinic and is therefore a biased sample. Also, all the scores were not cross-validated. All these raise the possibility of the findings being compromised. Conversely, a strength of the study is that the sample was large, and the data generated from this sample came from parents located in different parts of Australia.

As will be clear, in terms of psychometric properties, the current study focused mainly on internal structure (the extent to which item interrelationships conform to the intended construct), and relations to other variables (the pattern of relationships of test scores to external variables as predicted by the intended construct). Apart from these properties, the *Standards* (AERA, APA, NCME, 2014) that *“*provide criteria for the development and evaluation of tests and testing practices and to provide guidelines for assessing the validity of interpretations of test scores for the intended test uses” (p. 1), suggest examination of other properties (Hawkins et al., 2020), as being needed for interpreting and using test scores. These include aspects such as test content (i.e., the relationship of the item themes, wording and format with the intended construct, including administration process), response processes (the cognitive processes and interpretation of items by respondents and users, as measured against the intended construct), and consequences of testing (intended and unintended consequences, as can be traced to a source of invalidity such as construct under-representation or construct-irrelevant variance) as being needed for interpreting and using test scores. As these were not covered in the current study, it could be argued that although we conducted a relatively comprehensive evaluation of the psychometric properties, our coverage still falls short of all the requirements specified in the *Standards*. Consequently, more studies are needed in this area aimed at evaluating these psychometric properties whilst concurrently addressing the limitations noted above.

### 5.5 Summary and concluding remarks

In summary, the findings obtained from parents of adolescents in the current study supported the 17-factor model. Moreover, all of the factors in this model showed acceptable reliability (alpha and omega coefficients, and discriminant and criterion validity). Overall, therefore the findings indicated acceptable psychometric properties for the CAPP-PRF. Given that a major feature of the CAPP is that it screens for criteria that closely resemble the diagnostic criteria of the Diagnostic and Statistical Manual of Mental Disorders–Fifth Edition (DSM-5: American Psychiatric Association, 2013), it can contribute to more reliable diagnoses, including the provision of differential diagnosis and comorbidity information (Langsford et al., 2014). Therefore, in light of the CAPP-PRF’s acceptable psychometric properties and other valuable clinical characteristics, it is deemed suitable for clinical and research use for the screening of DSM-5 disorders among adolescents.

## Data availability statement

The raw data supporting the conclusions of this article will be made available by the first author, SL (shane@registeredpsychologist.com.au), upon reasonable request.

## Ethics statement

Ethical approval was not required for the study involving human samples in accordance with the local legislation and institutional requirements. Written informed consent for participation in this study was provided by the participants’ legal guardians/next of kin.

## Author contributions

SL: Conceptualization, Data curation, Methodology, Writing – original draft, Writing – review & editing. RG: Conceptualization, Formal analysis, Methodology, Writing – original draft, Writing – review & editing. SH: Writing – original draft, Writing – review & editing. LK: Conceptualization, Methodology, Validation, Writing – original draft, Writing – review & editing.

## Footnotes

^1^ www.psychprofiler.com
