# Supplementary material for: Psychometric properties of the Child and Adolescent PsychProfiler v5: a measure for screening 14 of the most common DSM-5 disorders
Source: Front Psychol. 2024 Aug 30;15:1267711. doi: 10.3389/fpsyg.2024.1267711 (PMC11394188; doi:10.3389/fpsyg.2024.1267711)
Supplement: Supplementary file 1 [file Data_Sheet_1.docx]

Supplementary Material

# Supplementary Data

The PsychProfiler v5 (Langsford, Houghton, & Douglas, 2014) is a comprehensive screening instrument for the simultaneous investigation of 20 of the most common disorders found in children, adolescents, and adults.

The Child and Adolescent PsychProfiler (CAPP) allows for the simultaneous screening of the 14 most prevalent disorders in children and adolescents, and the Adult PsychProfiler (APP) provides simultaneous screening of the 17 most prevalent disorders in adults.

The DSM-5 disorders in both the CAPP and APP are provided below underneath their respective DSM-5 disorder category (1 = included in the CAPP; 2 = included in the APP)

Supplementary Table S1

**Anxiety Disorders:**

✯ Generalised Anxiety Disorder (1 2)

✯ Panic Disorder (2)

✯ Separation Anxiety Disorder (1)

✯ Specific Phobia (2)

**Attention-Deficit/Hyperactivity Disorder:**

✯ Attention-Deficit/Hyperactivity Disorder (1 2)

**Autism Spectrum Disorder:**

✯ Autism Spectrum Disorder (1 2)

**Bipolar and Related Disorders:**

✯ Bipolar Disorder (2)

**Communication Disorders:**

✯ Language Disorder (1 2)

✯ Speech Sound Disorder (1 2)

**Depressive Disorders:**

✯ Persistent Depressive Disorder (1 2)

✯ Major Depressive Disorder (2)

**Disruptive, Impulse-Control, & Conduct Disorders:**

✯ Conduct Disorder (1)

✯ Oppositional Defiant Disorder (1)

**Feeding and Eating Disorders:**

✯ Anorexia Nervosa (1 2)

✯ Bulimia Nervosa (1 2)

**Obsessive-Compulsive and Related Disorders:**

✯ Obsessive-Compulsive Disorder (1 2)

**Personality Disorders:**

✯ Antisocial Personality Disorder (2)

**Schizophrenia Spectrum and Other Psychotic Disorders:**

✯ Schizophrenia (2)

**Specific Learning Disorders:**

✯ Specific Learning Disorder (1 2)

**Trauma and Stressor-Related Disorders:**

✯ Posttraumatic Stress Disorder (1 2)

**THEORETICAL BASIS OF THE CAPP**

Given the serious consequences associated with many *child and adolescent* ***disorders*** (i.e., specific individual disorders), knowledge about the prevalence of disorders within this population is important (Lewinsohn, Hops, Roberts, Seely, & Andrews, 1993). For example, disorders of depression have been shown to predict problems in the areas of school drop-out, unemployment, drug involvement, delinquent behaviour, and criminal conviction (Carlson & Stroeber, 1979; Chiles, Miller, & Cox, 1980; Kandel & Davies, 1986; Newcomb & Bentler, 1988; Paton, Kessler, & Kandel, 1977). Therefore, it is not surprising that a fairly large number of prevalence studies have been conducted. Unfortunately, many of the studies that have attempted to investigate prevalence have produced remarkable inconsistencies. One of the major reasons for this variation in prevalence studies is the inconsistency in the way in which diagnostic criteria are used, and hence, the confusion surrounding what actually constitutes a disorder.

**Definition of disorder:**

There is neither a clear definition of the term *disorder* that is universally satisfactory, nor a uniformly agreed upon method for ascertaining the existence of a disorder. From a medical point of view, disorders are perceived of as medical diseases, and the separate existence of disorders can be established by clinical description, epidemiology, premorbid personality, course, family history, laboratory findings, and response to treatment. More relevant to psychological and behavioural domains is construct validation including the description of diverse developmental pathways to ultimate symptom constructs. Without an agreed upon definition of disorder, the field appears to be moving on by using reliable criterion sets (Clarkin & Kendall, 1992; Hinkle, 1994; Kaslow, 1993; Wakefield, 1992) such as those provided in the widely used Diagnostic and Statistical Manual of Mental Disorders published by the American Psychiatric Association (DSM I-IV: APA, 1951-1994).

The Diagnostic and Statistical Manual of Mental Disorders (DSM-IV: APA, 1994, p. xxi) conceptualises disorder as:

*a clinically significant behavioural or psychological syndrome or pattern that occurs in an individual and that is associated with present distress (e.g., a painful symptom) or disability (i.e., impairment in one or more important areas of functioning) or with a significantly increased risk of suffering death, pain, disability, or an important loss of freedom.*

Despite this lengthy and comprehensive definition, the DSM-IV (APA, 1994, p. xxi) acknowledges that "although this manual provides a classification of mental disorders, it must be admitted that no definition adequately specifies precise boundaries for the concept of mental disorder". This lack of confidence in a definition of disorder is of particular importance to the DSM's conceptual structure because according to Wakefield (1992, p. 232) the DSM is "designed to be an atheoretical manual that is acceptable to clinicians and researchers of many theoretical persuasions".

**Methodological inconsistencies:**

Another of the major reasons for the inconsistency of results across studies is due to the different methodologies employed. Such studies have used a wide array of diagnostic criteria (many of which are now obsolete), including semistructured interviews and behavioural checklists. Understandably, the different instrumentation is likely to produce varying results, with the semistructured interview consistently being found to report the lower prevalence estimates (McGee et al., 1990). Furthermore, due to the variation in time commitments associated with the different diagnostic criteria, many studies investigating the prevalence of *child and adolescent* ***disorder*** (i.e., the collective grouping of individual disorders to include all children and adolescents with at least one disorder) have been forced to limit the number of disorders investigated (McGee et al., 1990). Therefore, many estimates of the prevalence of child and adolescent disorder have been made based on the investigation of only a small number of disorders. It seems likely therefore, that the prevalence of child and adolescent disorder is greater than that predicted on the basis of only three or four disorders.

In addition, many of the diagnostic methodologies have used parents and teachers as the source of information for assessing disorder in an individual. Obviously, adult perceptions will continue to be important, because adults are the source of child referrals. However, although traditionally child and adolescent's ratings of their own behaviour have been viewed as suspect (Hughes, 1988), their perceptions have now come to be considered valuable in their own right (Angold, 1996; Finch & Rogers, 1984; Mash & Terdal, 1988; Rohrbeck, Azar, & Wagner, 1991).

There are several reasons why the child or adolescent's self-report of his or her own behaviour might be useful. First, adult perceptions may be misperceptions, therefore, it is important to illuminate the unique information gained from a self-report measure, as compared to an adult rating of the child's behaviour. Second, unlike parent and teacher-reports, self-reports are more likely to reflect behaviour across all situations, not just the home or classroom (Beitchman & Corradini, 1988; Karoly, 1981). For example, parent-reports can be biased due to their subjectivity (Achenbach & Edelbrock, 1978; Barkley, 1981), whereas teacher-reports may reflect a child's behaviour in only one structured situation (e.g., mathematics class), or may reflect an idiosyncratic teacher-pupil relationship (Rohrbeck et al., 1991). Third, to exclude self-report perspectives may unnecessarily narrow the focus of interventions to observable behaviours and external contingencies. This could easily cause an under-reporting of symptoms that are more closely linked to internal emotional states, such as anxiety and depression (Weinstein, Noam, Grimes, Stone, & Schwab-Stone, 1990).

Another reason for the variation in results across prevalence studies is due to the differing characteristics of the samples. In the majority of cases, population prevalence estimates have been extrapolated from clinic samples. Only community samples reveal the true pattern of disorders, free from any self-selection into, or referral within the health care system. Furthermore, the age, gender, and number of participants have varied markedly, with many sample sizes often being too small to yield stable results (Lewinsohn et al., 1993).

The majority of prevalence studies have been conducted with adolescent participants (aged 13-17 years), and therefore information about disorder in children (aged 6-12 years) is sparse. The data available suggest that there is a slight rise in the rate of child and adolescent disorder between late childhood and early adolescence (Rutter, Tizard, Yule, Graham, & Whitmore, 1976). In accord, Tongue (1998) believes that the prevalence of child and adolescent disorder increases by 3-4% after puberty. Therefore, studies that have extrapolated the prevalence rates of childhood disorders from a sample of adolescents may well be over-reporting. However, Rutter et al. (1976) found approximately 40% of disorders seen in adolescence had persisted since childhood and the rest had newly developed during early adolescence. Similarly, up to one half of adult cases of obsessive-compulsive disorder (OCD) have been found to begin by the age of 15 years (Rapoport, 1986) and patients with eating disorders (EDs) typically report that their symptoms began during adolescence (Garfinkel & Garner, 1982). These findings highlight the need for future prevalence surveys to cover a wide age range of children and adolescents in order to extend our knowledge of the distribution, correlates, and typical age of onset of disorders (Offord, 1985). This developmental aspect needs to be addressed because the data resulting from such studies enable population trends to be accurately predicted, thereby making it feasible to plan future service needs in a more rational fashion.

The study of gender differences has been identified as an important area of child and adolescent disorder research because it is hoped that this type of research may lead eventually to a better understanding of the nature of disorders in males and females, concerning both similarities and differences. To date, studies have indicated that almost all disorders (except disorders such as anxiety, depression, and eating) are more common in males than in females. For example, learning disorders (LDs) have been found to be more common among males than females, with ratios ranging from 3:1 to 15:1 (Finucci & Childs, 1981). For this reason, the majority of research samples have been all male (or predominantly male). Because studies frequently focus on male participants very little is known about females and even less about gender differences (Vogel, 1990). Knowledge of how males and females differ might lead to the development of more appropriate screening and identification procedures and effective instructional strategies for females as distinct from those used with males.

Other methodological considerations exist because the prevalence of child and adolescent disorder has been found to be greater in low SES areas than in high SES areas (Bruce, Takeuchi, & Leaf, 1991; Holzer et al., 1986; Kessler et al., 1994; Tongue, 1998; Weich, Churchill, Lewis, & Mann, 1997; Williams, Takeuchi, & Adair, 1992). Furthermore, SES has been found to be associated not only with the onset, but also with the course of a disorder (Kessler et al., 1994). Unfortunately, almost all of the research pertaining to SES comprises adult participants. It is merely presumed that SES exerts the same influence on child and adolescent disorders.

**Prevalence of child and adolescent disorder:**

As a result of these differences in methodology, studies that have investigated the prevalence of child and adolescent disorder, have produced markedly varied results. Regier and Robins (1991) reported that the prevalence rates of child and adolescent disorder have been found to range from 11% to 57%. Despite estimates of prevalence reaching as high as 57%, a review of 25 studies (published between 1928 and 1974) by Gould, Wunsch-Hitzig, and Dohrenwend (1981) led the authors to estimate the prevalence of child and adolescent disorder to be 11.8%. However, more recent studies have consistently produced prevalence rates in excess of 20% (e.g., Fergusson, Horwood, & Lynskey, 1993; McGee et al., 1990; Merikangas et al., 2010; Department of Health and Ageing, 2013) with some reporting prevalence rates as high as 48% (Kessler et al., 1994). Similar variations are also reported for individual disorders. For example, the prevalence of attention-deficit/hyperactivity disorder (ADHD) has been reported from as low as 1% (McGee, Feehan, Williams, & Anderson, 1992) to as high as 13% (Velez, Johnson, & Cohen, 1989).

Although many of the studies that have reported the higher prevalence findings have been strongly criticised, the *true* prevalence rate of child and adolescent disorder may in fact be higher than that being commonly reported. A study by Costello et al. (1988) compared the prevalence rate of disorder diagnosed using a structured interview (22%) with that identified by paediatricians (5.7%). Furthermore, Costello et al. (1988) suggested that there exists among children and adolescents a large pool of disorders, and that these are largely unrecognised even by physicians who see the children regularly. Similarly, Prizant, et al. (1990, p. 186) stated that "current experience in our clinical setting and that reported in other settings for children with emotional and behavioural disorders (e.g., Cantwell, 1987) indicate that many children and adolescents with more subtle forms of disorder often are not identified". Therefore, it appears that the majority of children and adolescents with disorder are failing to be identified (Anderson, Williams, McGee, & Silva, 1987; Costello, 1989b; Costello et al., 1988; Offord et al., 1987). This is commensurate with the findings of studies from several industrialised countries which indicate that only a small proportion of children and adolescents with disorder actually receive specialist treatment (Burd, Kerbeshian, Cook, Bornhoeft, & Fisher, 1988; Costello, et al., 1988; Costello & Janiszewski, 1990; Offord et al., 1987; Merikangas et al., 2009; Brauner & Stephens, 2006). For example, Offord et al. (1987) found evidence of a disorder in 18.1% of children, but of these, only 6.5% had received any services from mental health or social service providers. Similarly, a study in New Zealand (NZ) reported that of the children and adolescents presenting with a disorder, only 21% were in contact with any service for their problems. (Fergusson et al., 1993).

This low incidence of identification, and hence low rate of treatment, may simply be due to the disorder(s) going undetected, or sometimes the result of a more overt disorder masking the more subtle disorders. For example, children with significant behavioural disorders (e.g., conduct disorder [CD]) who also experience a communication disorder (ComD) from early in the school years are often not identified as having a ComD. They may be placed in classes for the behaviourally disordered or emotionally disturbed, and because symptomatology of emotional and behavioural disorders are most often the primary concern, more subtle communication problems that may be involved directly in the development or perpetuation of the behavioural and/or emotional disturbance may be overlooked (Prizant et al., 1990). Similarly, Burd, Kauffman, and Kerbeshian (1992) found that less than 25% of children with Tourette's disorder (TD) had their LDs diagnosed in a school setting. Thus, ongoing identification is essential to provide appropriate services for children and adolescents with disorders that may simply go undetected during the school years or those with disorders that are masked by a more overt disorder.

**Comorbidity among disorders:**

In recent years, there has been a renewed interest and re-emphasis on the *comorbidity* among disorders. At its simplest, comorbidity is the occurrence at one point in time of two or more disorders (Clarkin & Kendall, 1992). The main purposes of collecting comorbidity data are to further our understanding of the disorders, to alert teachers and psychologists to possible complications, and to provide indications for treatment, prognosis, and educational service delivery (Rachman, 1991). Psychological analyses of comorbidity would serve each of these aims, and also open the way for more precise analyses of the relationship between two or more disorders. It would also provide a tool for determining whether or not any reported co-occurrence is a static association or a dynamic connection (Rachman, 1991).

Evidence of comorbidity among varying disorders has been well documented. Beitchmen, Nair, Clegg, and Patel (1986) conducted a study of five-year-old kindergarten children who were assessed for speech and language disorders. They found 11% had speech and language disorders, and of these, 48.7% presented with a disorder in addition to the speech and language disorder. In studies that have directly assessed the relationship between IQ and academic achievement, LDs have been found to occur in about 50% of children with TD (Bornstein, Carroll, & King, 1985; Burd et al., 1988; Golden, 1984; Hagin, Beecher, Pagano, & Kreeger, 1982; Hagin & Kugler, 1988; Joschko & Rourke, 1982). Similarly, there is a higher incidence of OCD in individuals with TD, with estimates ranging from approximately 35% to 50% (APA, 1994).

On the basis of prevalence studies and the results from specific populations, comorbidity among disorders can be considered extensive. Comorbidity has been found to be so extensive that some researchers (e.g., Blashfield, 1990; Brown & Barlow, 1992) have begun to question the assumption that the DSM-III-R (APA, 1987) has definite, discrete, and separate entities. This extensive comorbidity can be interpreted as suggesting that a dimensional approach to classification, rather than the categorical approach, might be the more parsimonious (Blashfield, 1990; Brown & Barlow, 1992; Kessler et al., 2012a). Therefore, it has been suggested that in addition to the simple determination of the co-occurrence of two disorders, attention should be given to the "psychological connectedness" (Rachman, 1991, p. 461) of the comorbid disorders and to consideration of "comorbid symptom clusters" (Clarkin & Kendall, 1992, p. 904).

In light of the Lewinsohn et al. (1993, p. 142) finding that "the degree of comorbidity between all of the major DSM-III-R disorders was found to be substantial", it is feasible that there is a single underlying factor common to all disorders. Researchers who have already attempted analyses from this theoretical standpoint include Beitchman (1985), Baker and Cantwell (1987b), and Baltaxe and Simmons (1988b). Unfortunately, to date, all research investigating the possibility of an underlying factor common to all disorders has proved inconclusive. Variables that have been examined include social class, low IQ, significant hearing loss, organic brain damage, adverse family conditions, low SES, mental retardation, marked hearing loss, brain damage, parental mental illness, family discord, and significant stress in childhood.

**Summary:**

Although a large number of prevalence studies have been conducted, to date there appears to have been very few investigations of disorder in mainstream children and adolescents in Australia. Of the few that have been conducted, the majority have been plagued by a number of methodological problems which has resulted in inconsistent findings.

# Supplementary Figures and Tables

Supplementary Table S2

Mean, Standard Deviation, and Dispersion Statistics of the 126 items of the PsychProfiler

| Items | Valid | Missing | Mean | Standard Deviation | Skewness | Kurtosis |
| --- | --- | --- | --- | --- | --- | --- |
| S112 | 932 | 19 | 2.56 | 1.58 | 0.09 | -1.02 |
| S1 | 936 | 15 | 2.87 | 1.46 | 0.03 | -1.04 |
| S94 | 939 | 12 | 1.82 | 1.44 | 0.52 | -0.51 |
| S54 | 941 | 10 | 2.06 | 1.44 | 0.52 | -0.54 |
| S15 | 913 | 38 | 1.72 | 1.45 | 0.55 | -0.56 |
| S24 | 940 | 11 | 2.36 | 1.72 | 0.22 | -1.20 |
| S118 | 928 | 23 | 2.72 | 1.58 | 0.03 | -1.06 |
| S69 | 940 | 11 | 2.00 | 1.57 | 0.48 | -0.79 |
| S64 | 935 | 16 | 0.74 | 1.19 | 1.87 | 3.14 |
| S23 | 910 | 41 | 1.13 | 1.42 | 1.28 | 0.80 |
| S111 | 918 | 33 | 0.83 | 1.27 | 1.72 | 2.33 |
| S70 | 925 | 26 | 1.14 | 1.52 | 1.28 | 0.58 |
| S36 | 929 | 22 | 1.08 | 1.40 | 1.39 | 1.20 |
| S43 | 928 | 23 | 1.03 | 1.47 | 1.42 | 0.99 |
| S51 | 902 | 49 | 0.54 | 0.95 | 2.27 | 5.82 |
| S18 | 937 | 14 | 1.11 | 1.47 | 1.29 | 0.72 |
| S46 | 943 | 8 | 1.84 | 1.75 | 0.51 | -1.06 |
| S79 | 939 | 12 | 1.69 | 1.67 | 0.71 | -0.71 |
| S30 | 942 | 9 | 1.04 | 1.30 | 1.35 | 1.16 |
| S74 | 941 | 10 | 1.47 | 1.51 | 0.88 | -0.19 |
| S72 | 938 | 13 | 1.68 | 1.46 | 0.79 | -0.21 |
| S40 | 940 | 11 | 1.79 | 1.52 | 0.69 | -0.48 |
| S13 | 931 | 20 | 1.71 | 1.47 | 0.68 | -0.43 |
| S87 | 927 | 24 | 1.42 | 1.43 | 0.97 | 0.21 |
| S50 | 942 | 9 | 2.13 | 1.62 | 0.43 | -0.94 |
| S57 | 939 | 12 | 3.04 | 1.49 | -0.17 | -1.03 |
| S81 | 935 | 16 | 2.43 | 1.44 | 0.27 | -0.73 |
| S104 | 942 | 9 | 2.47 | 1.50 | 0.23 | -0.96 |
| S75 | 937 | 14 | 2.63 | 1.47 | 0.13 | -0.90 |
| S100 | 940 | 11 | 2.98 | 1.57 | -0.23 | -1.04 |
| S3 | 948 | 3 | 3.48 | 1.49 | -0.64 | -0.64 |
| S42 | 942 | 9 | 2.65 | 1.60 | 0.10 | -1.17 |
| S97 | 938 | 13 | 3.12 | 1.55 | -0.29 | -1.07 |
| S6 | 945 | 6 | 2.83 | 1.49 | 0.02 | -1.09 |
| S35 | 940 | 11 | 1.52 | 1.62 | 0.84 | -0.44 |
| S5 | 947 | 4 | 1.98 | 1.65 | 0.52 | -0.90 |
| S116 | 935 | 16 | 1.44 | 1.47 | 0.96 | 0.03 |
| S71 | 934 | 17 | 1.65 | 1.54 | 0.74 | -0.44 |
| S8 | 942 | 9 | 2.02 | 1.59 | 0.49 | -0.81 |
| S123 | 938 | 13 | 1.64 | 1.52 | 0.77 | -0.33 |
| S59 | 937 | 14 | 0.86 | 1.35 | 1.72 | 2.12 |
| S124 | 862 | 89 | 0.89 | 1.23 | 1.75 | 2.69 |
| S126 | 924 | 27 | 1.26 | 1.62 | 1.14 | 0.06 |
| S41 | 940 | 11 | 2.07 | 1.64 | 0.45 | -0.95 |
| S82 | 938 | 13 | 0.80 | 1.34 | 1.79 | 2.33 |
| S62 | 932 | 19 | 1.25 | 1.59 | 1.13 | 0.07 |
| S11 | 943 | 8 | 1.18 | 1.39 | 1.12 | 0.45 |
| S115 | 934 | 17 | 1.75 | 1.66 | 0.66 | -0.77 |
| S114 | 937 | 14 | 2.11 | 1.50 | 0.49 | -0.66 |
| S32 | 941 | 10 | 1.89 | 1.59 | 0.53 | -0.78 |
| S78 | 937 | 14 | 1.23 | 1.46 | 1.12 | 0.34 |
| S109 | 937 | 14 | 1.64 | 1.47 | 0.76 | -0.26 |
| S125 | 933 | 18 | 2.37 | 1.54 | 0.27 | -0.93 |
| S84 | 941 | 10 | 1.77 | 1.49 | 0.61 | -0.48 |
| S68 | 940 | 11 | 0.88 | 1.36 | 1.66 | 1.89 |
| S48 | 905 | 46 | 0.77 | 1.23 | 1.89 | 3.19 |
| S122 | 936 | 15 | 0.92 | 1.34 | 1.63 | 1.95 |
| S83 | 938 | 13 | 0.82 | 1.22 | 1.67 | 2.35 |
| S91 | 930 | 21 | 1.80 | 1.48 | 0.67 | -0.40 |
| S107 | 940 | 11 | 2.50 | 1.58 | 0.19 | -1.06 |
| S28 | 936 | 15 | 2.43 | 1.57 | 0.21 | -1.02 |
| S38 | 934 | 17 | 2.68 | 1.60 | 2.66e-3 | -1.13 |
| S27 | 928 | 23 | 2.29 | 1.56 | 0.31 | -0.93 |
| S19 | 935 | 16 | 1.47 | 1.47 | 0.90 | -0.01 |
| S37 | 941 | 10 | 3.10 | 1.43 | -0.20 | -0.93 |
| S99 | 935 | 16 | 2.16 | 1.66 | 0.36 | -1.03 |
| S63 | 925 | 26 | 0.65 | 1.28 | 2.18 | 3.95 |
| S44 | 935 | 16 | 0.21 | 0.73 | 4.31 | 20.19 |
| S53 | 937 | 14 | 1.82 | 1.56 | 0.71 | -0.45 |
| S4 | 943 | 8 | 0.89 | 1.25 | 1.50 | 1.65 |
| S90 | 939 | 12 | 0.51 | 0.97 | 2.28 | 5.69 |
| S17 | 943 | 8 | 0.57 | 1.06 | 2.31 | 5.59 |
| S73 | 934 | 17 | 0.46 | 1.02 | 2.68 | 7.37 |
| S16 | 938 | 13 | 0.65 | 1.07 | 1.98 | 4.08 |
| S77 | 934 | 17 | 0.26 | 0.76 | 3.79 | 16.16 |
| S89 | 934 | 17 | 0.23 | 0.77 | 4.27 | 20.11 |
| S31 | 941 | 10 | 0.12 | 0.50 | 5.22 | 32.65 |
| S95 | 937 | 14 | 0.58 | 1.03 | 2.16 | 5.04 |
| S113 | 937 | 14 | 2.26 | 1.53 | 0.41 | -0.82 |
| S102 | 940 | 11 | 2.31 | 1.62 | 0.35 | -1.00 |
| S26 | 942 | 9 | 2.21 | 1.53 | 0.44 | -0.75 |
| S80 | 938 | 13 | 1.62 | 1.56 | 0.79 | -0.35 |
| S110 | 940 | 11 | 2.07 | 1.65 | 0.43 | -0.94 |
| S92 | 938 | 13 | 2.48 | 1.51 | 0.20 | -0.91 |
| S20 | 940 | 11 | 2.09 | 1.54 | 0.44 | -0.77 |
| S14 | 945 | 6 | 2.01 | 1.62 | 0.49 | -0.84 |
| S86 | 932 | 19 | 0.43 | 0.94 | 2.95 | 9.70 |
| S105 | 931 | 20 | 1.05 | 1.51 | 1.42 | 0.96 |
| S67 | 923 | 28 | 0.63 | 1.23 | 2.26 | 4.47 |
| S61 | 924 | 27 | 1.25 | 1.51 | 1.16 | 0.34 |
| S21 | 939 | 12 | 0.39 | 0.92 | 3.05 | 10.17 |
| S29 | 936 | 15 | 1.40 | 1.43 | 0.96 | 0.16 |
| S45 | 939 | 12 | 0.68 | 1.22 | 2.04 | 3.58 |
| S9 | 940 | 11 | 1.44 | 1.57 | 0.95 | -0.14 |
| S93 | 925 | 26 | 1.10 | 1.50 | 1.32 | 0.68 |
| S52 | 938 | 13 | 0.53 | 1.05 | 2.41 | 5.91 |
| S55 | 901 | 50 | 1.65 | 1.58 | 0.75 | -0.48 |
| S22 | 899 | 52 | 1.30 | 1.47 | 1.05 | 0.23 |
| S60 | 879 | 72 | 1.28 | 1.47 | 1.04 | 0.12 |
| S76 | 932 | 19 | 0.68 | 1.31 | 2.09 | 3.42 |
| S119 | 922 | 29 | 1.11 | 1.44 | 1.31 | 0.82 |
| S65 | 931 | 20 | 1.00 | 1.43 | 1.43 | 1.11 |
| S66 | 939 | 12 | 1.82 | 1.78 | 0.57 | -1.04 |
| S88 | 935 | 16 | 2.17 | 1.62 | 0.32 | -0.99 |
| S25 | 936 | 15 | 2.20 | 1.89 | 0.24 | -1.42 |
| S121 | 925 | 26 | 1.22 | 1.75 | 1.17 | -0.10 |
| S34 | 935 | 16 | 2.92 | 1.77 | -0.26 | -1.28 |
| S106 | 927 | 24 | 2.59 | 1.70 | -1.83e-3 | -1.23 |
| S58 | 942 | 9 | 2.41 | 1.72 | 0.24 | -1.24 |
| S96 | 939 | 12 | 2.50 | 1.80 | 0.08 | -1.34 |
| S108 | 920 | 31 | 1.48 | 1.81 | 0.86 | -0.72 |
| S47 | 934 | 17 | 2.57 | 1.77 | 0.05 | -1.32 |
| S103 | 925 | 26 | 2.19 | 1.75 | 0.31 | -1.16 |
| S12 | 936 | 15 | 0.88 | 1.28 | 1.65 | 2.25 |
| S56 | 931 | 20 | 2.33 | 1.83 | 0.21 | -1.35 |
| S33 | 929 | 22 | 1.96 | 1.84 | 0.41 | -1.24 |
| S2 | 923 | 28 | 1.65 | 1.53 | 0.72 | -0.47 |
| S98 | 911 | 40 | 1.48 | 1.46 | 0.88 | -0.06 |
| S7 | 913 | 38 | 0.93 | 1.22 | 1.51 | 1.89 |
| S85 | 893 | 58 | 1.07 | 1.36 | 1.29 | 0.94 |
| S10 | 923 | 28 | 1.64 | 1.59 | 0.76 | -0.50 |
| S120 | 908 | 43 | 0.93 | 1.38 | 1.53 | 1.47 |
| S49 | 933 | 18 | 2.10 | 1.53 | 0.42 | -0.79 |

Supplementary Table S3

Factor Loadings, and Omega Coefficient of the Seventeen-Factor PsychProfiler Model

|  | GAD | SAD | ADHDHI | ADHDI | ASD | LD | SSD | PDD | CD | ODD | AN | BN | OCD | SLDR | SLDW | SLDM | PTSD |
| --- | --- | --- | --- | --- | --- | --- | --- | --- | --- | --- | --- | --- | --- | --- | --- | --- | --- |
| S112 | .64 |  |  |  |  |  |  |  |  |  |  |  |  |  |  |  |  |
| S1 | .66 |  |  |  |  |  |  |  |  |  |  |  |  |  |  |  |  |
| S94 | .62 |  |  |  |  |  |  |  |  |  |  |  |  |  |  |  |  |
| S54 | .69 |  |  |  |  |  |  |  |  |  |  |  |  |  |  |  |  |
| S15 | .51 |  |  |  |  |  |  |  |  |  |  |  |  |  |  |  |  |
| S24 | .59 |  |  |  |  |  |  |  |  |  |  |  |  |  |  |  |  |
| S118 | .39 |  |  |  |  |  |  |  |  |  |  |  |  |  |  |  |  |
| S69 | .59 |  |  |  |  |  |  |  |  |  |  |  |  |  |  |  |  |
| S64 |  | .76 |  |  |  |  |  |  |  |  |  |  |  |  |  |  |  |
| S23 |  | .73 |  |  |  |  |  |  |  |  |  |  |  |  |  |  |  |
| S111 |  | .84 |  |  |  |  |  |  |  |  |  |  |  |  |  |  |  |
| S70 |  | .56 |  |  |  |  |  |  |  |  |  |  |  |  |  |  |  |
| S36 |  | .80 |  |  |  |  |  |  |  |  |  |  |  |  |  |  |  |
| S43 |  | .77 |  |  |  |  |  |  |  |  |  |  |  |  |  |  |  |
| S51 |  | .73 |  |  |  |  |  |  |  |  |  |  |  |  |  |  |  |
| S18 |  | .74 |  |  |  |  |  |  |  |  |  |  |  |  |  |  |  |
| S46 |  |  | .62 |  |  |  |  |  |  |  |  |  |  |  |  |  |  |
| S79 |  |  | .75 |  |  |  |  |  |  |  |  |  |  |  |  |  |  |
| S30 |  |  | .70 |  |  |  |  |  |  |  |  |  |  |  |  |  |  |
| S74 |  |  | .80 |  |  |  |  |  |  |  |  |  |  |  |  |  |  |
| S72 |  |  | .44 |  |  |  |  |  |  |  |  |  |  |  |  |  |  |
| S40 |  |  | .61 |  |  |  |  |  |  |  |  |  |  |  |  |  |  |
| S13 |  |  | .69 |  |  |  |  |  |  |  |  |  |  |  |  |  |  |
| S87 |  |  | .73 |  |  |  |  |  |  |  |  |  |  |  |  |  |  |
| S50 |  |  | .74 |  |  |  |  |  |  |  |  |  |  |  |  |  |  |
| S57 |  |  |  | .85 |  |  |  |  |  |  |  |  |  |  |  |  |  |
| S81 |  |  |  | .67 |  |  |  |  |  |  |  |  |  |  |  |  |  |
| S104 |  |  |  | .82 |  |  |  |  |  |  |  |  |  |  |  |  |  |
| S75 |  |  |  | .74 |  |  |  |  |  |  |  |  |  |  |  |  |  |
| S100 |  |  |  | .77 |  |  |  |  |  |  |  |  |  |  |  |  |  |
| S3 |  |  |  | .66 |  |  |  |  |  |  |  |  |  |  |  |  |  |
| S42 |  |  |  | .72 |  |  |  |  |  |  |  |  |  |  |  |  |  |
| S97 |  |  |  | .84 |  |  |  |  |  |  |  |  |  |  |  |  |  |
| S6 |  |  |  | .68 |  |  |  |  |  |  |  |  |  |  |  |  |  |
| S35 |  |  |  |  | .82 |  |  |  |  |  |  |  |  |  |  |  |  |
| S5 |  |  |  |  | .58 |  |  |  |  |  |  |  |  |  |  |  |  |
| S116 |  |  |  |  | .55 |  |  |  |  |  |  |  |  |  |  |  |  |
| S71 |  |  |  |  | .67 |  |  |  |  |  |  |  |  |  |  |  |  |
| S8 |  |  |  |  | .72 |  |  |  |  |  |  |  |  |  |  |  |  |
| S123 |  |  |  |  | .67 |  |  |  |  |  |  |  |  |  |  |  |  |
| S59 |  |  |  |  | .71 |  |  |  |  |  |  |  |  |  |  |  |  |
| S124 |  |  |  |  | .49 |  |  |  |  |  |  |  |  |  |  |  |  |
| S126 |  |  |  |  | .56 |  |  |  |  |  |  |  |  |  |  |  |  |
| S41 |  |  |  |  | .61 |  |  |  |  |  |  |  |  |  |  |  |  |
| S82 |  |  |  |  | .48 |  |  |  |  |  |  |  |  |  |  |  |  |
| S62 |  |  |  |  | .61 |  |  |  |  |  |  |  |  |  |  |  |  |
| S11 |  |  |  |  |  | .72 |  |  |  |  |  |  |  |  |  |  |  |
| S115 |  |  |  |  |  | .82 |  |  |  |  |  |  |  |  |  |  |  |
| S114 |  |  |  |  |  | .78 |  |  |  |  |  |  |  |  |  |  |  |
| S32 |  |  |  |  |  | .60 |  |  |  |  |  |  |  |  |  |  |  |
| S78 |  |  |  |  |  | .81 |  |  |  |  |  |  |  |  |  |  |  |
| S109 |  |  |  |  |  | .82 |  |  |  |  |  |  |  |  |  |  |  |
| S125 |  |  |  |  |  | .77 |  |  |  |  |  |  |  |  |  |  |  |
| S84 |  |  |  |  |  | .74 |  |  |  |  |  |  |  |  |  |  |  |
| S68 |  |  |  |  |  |  | .85 |  |  |  |  |  |  |  |  |  |  |
| S48 |  |  |  |  |  |  | .77 |  |  |  |  |  |  |  |  |  |  |
| S122 |  |  |  |  |  |  | .89 |  |  |  |  |  |  |  |  |  |  |
| S83 |  |  |  |  |  |  | .80 |  |  |  |  |  |  |  |  |  |  |
| S91 |  |  |  |  |  |  |  | .71 |  |  |  |  |  |  |  |  |  |
| S107 |  |  |  |  |  |  |  | .75 |  |  |  |  |  |  |  |  |  |
| S28 |  |  |  |  |  |  |  | .64 |  |  |  |  |  |  |  |  |  |
| S38 |  |  |  |  |  |  |  | .71 |  |  |  |  |  |  |  |  |  |
| S27 |  |  |  |  |  |  |  | .72 |  |  |  |  |  |  |  |  |  |
| S19 |  |  |  |  |  |  |  | .52 |  |  |  |  |  |  |  |  |  |
| S37 |  |  |  |  |  |  |  | .47 |  |  |  |  |  |  |  |  |  |
| S99 |  |  |  |  |  |  |  | .62 |  |  |  |  |  |  |  |  |  |
| S63 |  |  |  |  |  |  |  |  | .37 |  |  |  |  |  |  |  |  |
| S44 |  |  |  |  |  |  |  |  | .33 |  |  |  |  |  |  |  |  |
| S53 |  |  |  |  |  |  |  |  | .59 |  |  |  |  |  |  |  |  |
| S4 |  |  |  |  |  |  |  |  | .81 |  |  |  |  |  |  |  |  |
| S90 |  |  |  |  |  |  |  |  | .80 |  |  |  |  |  |  |  |  |
| S17 |  |  |  |  |  |  |  |  | .61 |  |  |  |  |  |  |  |  |
| S73 |  |  |  |  |  |  |  |  | .66 |  |  |  |  |  |  |  |  |
| S16 |  |  |  |  |  |  |  |  | .78 |  |  |  |  |  |  |  |  |
| S77 |  |  |  |  |  |  |  |  | .56 |  |  |  |  |  |  |  |  |
| S89 |  |  |  |  |  |  |  |  | .68 |  |  |  |  |  |  |  |  |
| S31 |  |  |  |  |  |  |  |  | .39 |  |  |  |  |  |  |  |  |
| S95 |  |  |  |  |  |  |  |  | .81 |  |  |  |  |  |  |  |  |
| S113 |  |  |  |  |  |  |  |  |  | .84 |  |  |  |  |  |  |  |
| S102 |  |  |  |  |  |  |  |  |  | .80 |  |  |  |  |  |  |  |
| S26 |  |  |  |  |  |  |  |  |  | .72 |  |  |  |  |  |  |  |
| S80 |  |  |  |  |  |  |  |  |  | .72 |  |  |  |  |  |  |  |
| S110 |  |  |  |  |  |  |  |  |  | .78 |  |  |  |  |  |  |  |
| S92 |  |  |  |  |  |  |  |  |  | .73 |  |  |  |  |  |  |  |
| S20 |  |  |  |  |  |  |  |  |  | .83 |  |  |  |  |  |  |  |
| S14 |  |  |  |  |  |  |  |  |  | .83 |  |  |  |  |  |  |  |
| S86 |  |  |  |  |  |  |  |  |  |  | .81 |  |  |  |  |  |  |
| S105 |  |  |  |  |  |  |  |  |  |  | .95 |  |  |  |  |  |  |
| S67 |  |  |  |  |  |  |  |  |  |  | .80 |  |  |  |  |  |  |
| S61 |  |  |  |  |  |  |  |  |  |  | .91 |  |  |  |  |  |  |
| S21 |  |  |  |  |  |  |  |  |  |  | .68 |  |  |  |  |  |  |
| S29 |  |  |  |  |  |  |  |  |  |  |  | .58 |  |  |  |  |  |
| S45 |  |  |  |  |  |  |  |  |  |  |  | .47 |  |  |  |  |  |
| S9 |  |  |  |  |  |  |  |  |  |  |  | .89 |  |  |  |  |  |
| S93 |  |  |  |  |  |  |  |  |  |  |  | .93 |  |  |  |  |  |
| S52 |  |  |  |  |  |  |  |  |  |  |  | .60 |  |  |  |  |  |
| S55 |  |  |  |  |  |  |  |  |  |  |  |  | .89 |  |  |  |  |
| S22 |  |  |  |  |  |  |  |  |  |  |  |  | .82 |  |  |  |  |
| S60 |  |  |  |  |  |  |  |  |  |  |  |  | .82 |  |  |  |  |
| S76 |  |  |  |  |  |  |  |  |  |  |  |  | .38 |  |  |  |  |
| S119 |  |  |  |  |  |  |  |  |  |  |  |  | .45 |  |  |  |  |
| S65 |  |  |  |  |  |  |  |  |  |  |  |  | .42 |  |  |  |  |
| S66 |  |  |  |  |  |  |  |  |  |  |  |  |  | .91 |  |  |  |
| S88 |  |  |  |  |  |  |  |  |  |  |  |  |  | .81 |  |  |  |
| S25 |  |  |  |  |  |  |  |  |  |  |  |  |  | .88 |  |  |  |
| S121 |  |  |  |  |  |  |  |  |  |  |  |  |  | .73 |  |  |  |
| S34 |  |  |  |  |  |  |  |  |  |  |  |  |  |  | .82 |  |  |
| S106 |  |  |  |  |  |  |  |  |  |  |  |  |  |  | .88 |  |  |
| S58 |  |  |  |  |  |  |  |  |  |  |  |  |  |  | .78 |  |  |
| S96 |  |  |  |  |  |  |  |  |  |  |  |  |  |  | .57 |  |  |
| S108 |  |  |  |  |  |  |  |  |  |  |  |  |  |  | .68 |  |  |
| S47 |  |  |  |  |  |  |  |  |  |  |  |  |  |  |  | .93 |  |
| S103 |  |  |  |  |  |  |  |  |  |  |  |  |  |  |  | .90 |  |
| S12 |  |  |  |  |  |  |  |  |  |  |  |  |  |  |  | .62 |  |
| S56 |  |  |  |  |  |  |  |  |  |  |  |  |  |  |  | .93 |  |
| S33 |  |  |  |  |  |  |  |  |  |  |  |  |  |  |  | .79 |  |
| S2 |  |  |  |  |  |  |  |  |  |  |  |  |  |  |  |  | .75 |
| S98 |  |  |  |  |  |  |  |  |  |  |  |  |  |  |  |  | .71 |
| S7 |  |  |  |  |  |  |  |  |  |  |  |  |  |  |  |  | .70 |
| S85 |  |  |  |  |  |  |  |  |  |  |  |  |  |  |  |  | .80 |
| S10 |  |  |  |  |  |  |  |  |  |  |  |  |  |  |  |  | .72 |
| S120 |  |  |  |  |  |  |  |  |  |  |  |  |  |  |  |  | .76 |
| S49 |  |  |  |  |  |  |  |  |  |  |  |  |  |  |  |  | .55 |
| Reliability | | | | | | | | | | | | | | | | | |
| Omega | .809 | .908 | .885 | .921 | .885 | .916 | .897 | .851 | .885 | .927 | .919 | .832 | .811 | .902 | .866 | .923 | .880 |
| Alpha | .802 | .899 | .887 | .922 | .884 | .916 | .898 | .851 | .872 | .928 | .876 | .759 | .830 | .894 | .860 | .919 | .872 |
|  | GAD | SAD | ADHDHI | ADHDI | ASD | LD | SSD | PDD | CD | ODD | AN | BN | OCD | SLDR | SLDW | SLDM | PTSD |

*Note*. GAD = Generalised Anxiety Disorder; SAD = Separation Anxiety Disorder, ADHDHI = Attention-Deficit/Hyperactivity Disorder: Hyperactive/Impulsive; ADHDI = Attention Deficit/Hyperactivity Disorder: Inattentive; ASD = Autism Spectrum Disorder; LD = Language Disorder; SSD =Speech Sound Disorder; PDD = Persistent Depressive Disorder; CD = Conduct Disorder; ODD = Oppositional Defiant Disorder; AN = Anorexia Nervosa; BN = Bulimia Nervosa; OCD = Obsessive-Compulsive Disorder; SLDR = Specific Learning Disorder: Reading; SLDW = Specific Learning Disorder: Written Expression; SLDM = Specific Learning Disorder: Mathematics; PTSD = Posttraumatic Stress Disorder.

Supplementary Table S4

Factor Correlations of the Factors in the Seventeen-Factor PsychProfiler Model

|  | 1 | 2 | 3 | 4 | 5 | 6 | 7 | 8 | 9 | 10 | 11 | 12 | 13 | 14 | 15 | 16 | 17 |
| --- | --- | --- | --- | --- | --- | --- | --- | --- | --- | --- | --- | --- | --- | --- | --- | --- | --- |
| GAD (1) | .- | .59 | .45 | .45 | .59 | .26 | .16 | 1.00 | .44 | .65 | .54 | .57 | .81 | .06 | .06 | .17 | .78 |
| SAD (2) |  | - | .35 | .23 | .42 | .24 | .21 | .46 | .23 | .28 | .30 | .32 | .55 | .16 | .15 | .19 | .62 |
| ADHDHI (3) |  |  | - | .71 | .58 | .45 | .37 | .38 | .57 | .67 | .13 | .19 | .27 | .30 | .38 | .21 | .36 |
| ADHDI (4) |  |  |  | - | .52 | .61 | .35 | .46 | .37 | .52 | .05 | .11 | .19 | .41 | .55 | .40 | .27 |
| ASD (5) |  |  |  |  | - | .61 | .50 | .59 | .49 | .56 | .23 | .27 | .45 | .25 | .34 | .23 | .54 |
| LD (6) |  |  |  |  |  | - | .77 | .28 | .25 | .28 | .10 | .11 | .15 | .77 | .77 | .55 | .25 |
| SSD (7) |  |  |  |  |  |  | - | .15 | .22 | .17 | .10 | .11 | .10 | .62 | .60 | .40 | .25 |
| PDD (8) |  |  |  |  |  |  |  | - | .45 | .68 | .60 | .65 | .71 | .09 | .10 | .19 | .70 |
| CD (9) |  |  |  |  |  |  |  |  | - | .80 | .26 | .29 | .34 | .13 | .14 | .13 | .39 |
| ODD (10) |  |  |  |  |  |  |  |  |  | - | .29 | .34 | .41 | .12 | .15 | .14 | .46 |
| AN (11) |  |  |  |  |  |  |  |  |  |  | - | 1.00 | .49 | .02 | -.05 | .11 | .50 |
| BN (12) |  |  |  |  |  |  |  |  |  |  |  | - | .48 | .02 | -.04 | .11 | .53 |
| OCD (13) |  |  |  |  |  |  |  |  |  |  |  |  | - | -.01 | -.05 | .10 | .73 |
| SLDR (14) |  |  |  |  |  |  |  |  |  |  |  |  |  | - | .86 | .53 | .09 |
| SLDW (15) |  |  |  |  |  |  |  |  |  |  |  |  |  |  | - | .54 | .06 |
| SLDM (16) |  |  |  |  |  |  |  |  |  |  |  |  |  |  |  | - | .16 |
| PTSD (17) |  |  |  |  |  |  |  |  |  |  |  |  |  |  |  |  | - |

*Note*. GAD = Generalised Anxiety Disorder; SAD = Separation Anxiety Disorder, ADHDHI = Attention-Deficit/Hyperactivity Disorder: Hyperactive/Impulsive; ADHDI = Attention Deficit/Hyperactivity Disorder: Inattentive; ASD = Autism Spectrum Disorder; LD = Language Disorder; SSD =Speech Sound Disorder; PDD = Persistent Depressive Disorder; CD = Conduct Disorder; ODD = Oppositional Defiant Disorder; AN = Anorexia Nervosa; BN = Bulimia Nervosa; OCD = Obsessive-Compulsive Disorder; SLDR = Specific Learning Disorder: Reading; SLDW = Specific Learning Disorder: Written Expression; SLDM = Specific Learning Disorder: Mathematics; PTSD = Posttraumatic Stress Disorder. Underlined correlations (< .05) are not significant

Supplementary Table S5

***Convergent and Discriminant Validity of the Factors in the Seventeen-Factor PsychProfiler Model in Terms of Relationships with the Conners 3-P Scales***

|  | DSM-5 symptom scales | | | | | | | |
| --- | --- | --- | --- | --- | --- | --- | --- | --- |
|  | C-ADHDI | | C-ADHDHI | | C-CD | | C-ODD | |
| PP Scales | r | r^2^ | r | r^2^ | r | r^2^ | r | r^2^ |
| GAD | 0.313 | 0.098 | 0.266 | 0.071 | 0.400 | 0.160 | 0.333 | 0.111 |
| SAD | 0.073 | 0.005 | 0.197 | 0.039 | 0.184 | 0.034 | 0.182 | 0.033 |
| ADHDHI | 0.212 | 0.045 | 0.575 | 0.331 | 0.229 | 0.052 | 0.295 | 0.087 |
| ADHDI | 0.679 | 0.461 | 0.459 | 0.211 | 0.212 | 0.045 | 0.272 | 0.074 |
| ASD | 0.149 | 0.022 | 0.19 | 0.036 | 0.115 | 0.013 | 0.163 | 0.027 |
| LD | 0.318 | 0.101 | 0.164 | 0.027 | 0.029 | 0.001 | 0.035 | 0.001 |
| SSD | 0.134 | 0.018 | -0.027 | 0.001 | 0.052 | 0.003 | -0.063 | 0.004 |
| PDD | 0.301 | 0.091 | 0.308 | 0.095 | 0.327 | 0.107 | 0.394 | 0.155 |
| CD | 0.171 | 0.029 | 0.352 | 0.124 | 0.626 | 0.392 | 0.579 | 0.335 |
| ODD | 0.131 | 0.017 | 0.192 | 0.037 | 0.113 | 0.013 | 0.214 | 0.046 |
| AN | 0.148 | 0.022 | 0.126 | 0.016 | 0.165 | 0.027 | 0.113 | 0.013 |
| BN | 0.132 | 0.017 | 0.109 | 0.012 | 0.333 | 0.111 | 0.251 | 0.063 |
| OCD | 0.05 | 0.003 | 0.049 | 0.002 | 0.142 | 0.020 | 0.084 | 0.007 |
| SLDR | 0.123 | 0.015 | 0.018 | 0.000 | -0.034 | 0.001 | -0.088 | 0.008 |
| SLDW | 0.189 | 0.036 | 0.133 | 0.018 | 0.039 | 0.002 | -0.056 | 0.003 |
| SLDM | 0.172 | 0.030 | 0.003 | 0.000 | 0.083 | 0.007 | -0.069 | 0.005 |
| PTSD | 0.177 | 0.031 | 0.271 | 0.073 | 0.309 | 0.095 | 0.204 | 0.042 |

Value r (correlation) ≥ .15 [or r^2^ (variance ≥ .023)] significant at *p* =.05..

*Note*. GAD = Generalised Anxiety Disorder; SAD = Separation Anxiety Disorder, ADHDHI = Attention-Deficit/Hyperactivity Disorder: Hyperactive/Impulsive; ADHDI = Attention Deficit/Hyperactivity Disorder: Inattentive; ASD = Autism Spectrum Disorder; LD = Language Disorder; SSD =Speech Sound Disorder; PDD = Persistent Depressive Disorder; CD = Conduct Disorder; ODD = Oppositional Defiant Disorder; AN = Anorexia Nervosa; BN = Bulimia Nervosa; OCD = Obsessive-Compulsive Disorder; SLDR = Specific Learning Disorder: Reading; SLDW = Specific Learning Disorder: Written Expression; SLDM = Specific Learning Disorder: Mathematics; PTSD = Posttraumatic Stress Disorder; C-ADHDI = Conners-3-P ADHD Inattentive ; C-ADHDHI = Conners-3-P ADHD Hyperactive/Impulsive; C-CD = Conners-3-P Conduct Disorder; C = ODD = Conners-3-P Oppositional Defiant Disorder.

Supplementary Table S6

***Convergent and Discriminant Validity of the Factors in the Seventeen-Factor PsychProfiler Model in Terms of Relationships with the BYI-2 Scales***

|  | BYI-2 Scales | | | | | | | | | |
| --- | --- | --- | --- | --- | --- | --- | --- | --- | --- | --- |
| PP Scale | SC | | Anx | | Dep | | Anger | | DB | |
|  | r | r^2^ | r | r^2^ | r | r^2^ | r | r^2^ | r | r^2^ |
| GAD | -0.387 | 0.150 | 0.348 | 0.121 | 0.384 | 0.147 | 0.403 | 0.162 | 0.356 | 0.127 |
| SAD | -0.065 | 0.004 | 0.118 | 0.014 | 0.124 | 0.015 | 0.277 | 0.077 | 0.155 | 0.024 |
| ADHDHI | 0.028 | 0.001 | -0.013 | 0.000 | -0.015 | 0.000 | 0.106 | 0.011 | 0.046 | 0.002 |
| ADHDI | -0.055 | 0.003 | 0.034 | 0.001 | -0.006 | 0.000 | 0.099 | 0.010 | 0.151 | 0.023 |
| ASD | -0.073 | 0.005 | -0.041 | 0.002 | 0.023 | 0.001 | 0.107 | 0.011 | 0.081 | 0.007 |
| LD | -0.084 | 0.007 | -0.055 | 0.003 | -0.057 | 0.003 | -0.002 | 0.000 | 0.077 | 0.006 |
| SSD | 0.028 | 0.001 | -0.023 | 0.001 | -0.083 | 0.007 | -0.028 | 0.001 | -0.014 | 0.000 |
| PDD | -0.447 | 0.200 | 0.367 | 0.135 | 0.418 | 0.175 | 0.480 | 0.230 | 0.384 | 0.147 |
| CD | -0.169 | 0.029 | 0.029 | 0.001 | 0.040 | 0.002 | 0.175 | 0.031 | 0.372 | 0.138 |
| ODD | -0.122 | 0.015 | -0.017 | 0.000 | 0.052 | 0.003 | 0.079 | 0.006 | 0.072 | 0.005 |
| AN | -0.207 | 0.043 | 0.197 | 0.039 | 0.225 | 0.051 | 0.198 | 0.039 | 0.218 | 0.048 |
| BN | -0.181 | 0.033 | 0.156 | 0.024 | 0.215 | 0.046 | 0.260 | 0.068 | 0.321 | 0.103 |
| OCD | -0.033 | 0.001 | -0.032 | 0.001 | -0.002 | 0.000 | 0.005 | 0.000 | -0.081 | 0.007 |
| SLDR | 0.078 | 0.006 | -0.129 | 0.017 | -0.170 | 0.029 | -0.125 | 0.016 | -0.019 | 0.000 |
| SLDW | 0.067 | 0.004 | -0.196 | 0.038 | -0.222 | 0.049 | -0.120 | 0.014 | -0.064 | 0.004 |
| SLDM | -0.045 | 0.002 | -0.099 | 0.010 | -0.104 | 0.011 | -0.047 | 0.002 | 0.066 | 0.004 |
| PTSD | -0.101 | 0.010 | 0.163 | 0.027 | 0.220 | 0.048 | 0.208 | 0.043 | 0.242 | 0.059 |

Value r (correlation) ≥ .15 [or r^2^ (variance ≥ .023)] significant at *p* =.05.

*Note*. GAD = Generalised Anxiety Disorder; SAD = Separation Anxiety Disorder, ADHDHI = Attention-Deficit/Hyperactivity Disorder: Hyperactive/Impulsive; ADHDI = Attention Deficit/Hyperactivity Disorder: Inattentive; ASD = Autism Spectrum Disorder; LD = Language Disorder; SSD =Speech Sound Disorder; PDD = Persistent Depressive Disorder; CD = Conduct Disorder; ODD = Oppositional Defiant Disorder; AN = Anorexia Nervosa; BN = Bulimia Nervosa; OCD = Obsessive-Compulsive Disorder; SLDR = Specific Learning Disorder: Reading; SLDW = Specific Learning Disorder: Written Expression; SLDM = Specific Learning Disorder: Mathematics; PTSD = Posttraumatic Stress Disorder; SC = Self-Concept; Anx =Anxiety; Dep = Depression; DB = Disruptive Behaviour; BYI-2 = Beck Youth Inventories, Second Edition,

Supplementary Table S7

***Criterion Validity of the Factors in the Seventeen-Factor PsychProfiler Model in Terms of Relationships with the WISC-V Composite Scores***

|  | WISC-V Composite Score | | | | | | | | | | | |
| --- | --- | --- | --- | --- | --- | --- | --- | --- | --- | --- | --- | --- |
|  | VCI | | VSI | | FRI | | WMI | | PSI | | FSIQ | |
|  | r | r^2^ | r | r^2^ | r | r^2^ | r | r^2^ | r | r^2^ | r | r^2^ |
| GAD | -0.045 | 0.002 | 0.023 | 0.001 | 0.048 | 0.002 | -0.001 | 0.000 | 0.010 | 0.000 | -0.046 | 0.002 |
| SAD | -0.058 | 0.003 | -0.037 | 0.001 | -0.029 | 0.001 | -0.016 | 0.000 | -0.065 | 0.004 | 0.021 | 0.000 |
| ADHDHI | 0.049 | 0.002 | 0.143 | 0.020 | 0.174 | 0.030 | 0.022 | 0.000 | 0.060 | 0.004 | 0.098 | 0.010 |
| ADHDI | -0.151 | 0.023 | 0.034 | 0.001 | 0.032 | 0.001 | -0.140 | 0.020 | -0.079 | 0.006 | -0.138 | 0.019 |
| ASD | 0.051 | 0.003 | 0.012 | 0.000 | -0.006 | 0.000 | 0.060 | 0.004 | 0.113 | 0.013 | 0.055 | 0.003 |
| LD | -0.217 | 0.047 | -0.089 | 0.008 | -0.106 | 0.011 | -0.125 | 0.016 | -0.060 | 0.004 | -0.114 | 0.013 |
| SSD | -0.130 | 0.017 | -0.060 | 0.004 | -0.080 | 0.006 | -0.121 | 0.015 | -0.132 | 0.017 | -0.223 | 0.050 |
| PDD | -0.060 | 0.004 | -0.011 | 0.000 | -0.005 | 0.000 | -0.005 | 0.000 | 0.047 | 0.002 | 0.033 | 0.001 |
| CD | 0.024 | 0.001 | -0.022 | 0.000 | -0.024 | 0.001 | 0.067 | 0.004 | -0.019 | 0.000 | 0.057 | 0.003 |
| ODD | -0.169 | 0.029 | -0.036 | 0.001 | -0.030 | 0.001 | -0.135 | 0.018 | -0.084 | 0.007 | -0.091 | 0.008 |
| AN | 0.047 | 0.002 | -0.001 | 0.000 | 0.004 | 0.000 | 0.074 | 0.005 | 0.142 | 0.020 | 0.032 | 0.001 |
| BN | -0.006 | 0.000 | -0.074 | 0.005 | -0.086 | 0.007 | 0.010 | 0.000 | 0.056 | 0.003 | -0.013 | 0.000 |
| OCD | -0.143 | 0.020 | -0.061 | 0.004 | -0.049 | 0.002 | -0.109 | 0.012 | -0.021 | 0.000 | -0.034 | 0.001 |
| SLDR | -0.167 | 0.028 | -0.023 | 0.001 | -0.036 | 0.001 | -0.141 | 0.020 | -0.136 | 0.018 | -0.140 | 0.020 |
| SLDW | -0.162 | 0.026 | -0.029 | 0.001 | -0.024 | 0.001 | -0.224 | 0.050 | -0.151 | 0.023 | -0.102 | 0.010 |
| SLDM | -0.261 | 0.068 | -0.084 | 0.007 | -0.072 | 0.005 | -0.255 | 0.065 | -0.232 | 0.054 | -0.223 | 0.050 |
| PTSD | -0.067 | 0.004 | -0.101 | 0.010 | -0.103 | 0.011 | -0.058 | 0.003 | -0.030 | 0.001 | -0.085 | 0.007 |

Value r (correlation) ≥ .15 [or r^2^ (variance ≥ .023)] significant at *p* =.05.

*Note*. GAD = Generalised Anxiety Disorder; SAD = Separation Anxiety Disorder, ADHDHI = Attention-Deficit/Hyperactivity Disorder: Hyperactive/Impulsive; ADHDI = Attention Deficit/Hyperactivity Disorder: Inattentive; ASD = Autism Spectrum Disorder; LD = Language Disorder; SSD =Speech Sound Disorder; PDD = Persistent Depressive Disorder; CD = Conduct Disorder; ODD = Oppositional Defiant Disorder; AN = Anorexia Nervosa; BN = Bulimia Nervosa; OCD = Obsessive-Compulsive Disorder; SLDR = Specific Learning Disorder: Reading; SLDW = Specific Learning Disorder: Written Expression; SLDM = Specific Learning Disorder: Mathematics; PTSD = Posttraumatic Stress Disorder; WISC-V = Wechsler Intelligence Scale for Children, Fifth Edition; VCI = Verbal Comprehension Index; VSI = Visual Spatial Index; FRI = Fluid Reasoning Index; WMI = Working Memory Index, PSI = Processing Speed Index; FSIQ = Full-Scale Intelligence Quotient.

Supplementary Table S8

***Criterion Validity of the Factors in the Seventeen-Factor PsychProfiler Model in Terms of Relationships with the WIAT-III Composite Scores***

|  | WIAT-III | | | | | |
| --- | --- | --- | --- | --- | --- | --- |
| PP Scale | Read | | Written | | Maths | |
|  | r | r^2^ | r | r^2^ | r | r^2^ |
| GAD | -0.205 | 0.042 | -0.133 | 0.018 | -0.065 | 0.004 |
| SAD | -0.140 | 0.020 | -0.143 | 0.020 | 0.046 | 0.002 |
| ADHDHI | -0.003 | 0.000 | -0.044 | 0.002 | -0.041 | 0.002 |
| ADHDI | -0.085 | 0.007 | -0.021 | 0.000 | 0.009 | 0.000 |
| ASD | -0.045 | 0.002 | -0.090 | 0.008 | -0.035 | 0.001 |
| LD | 0.165 | 0.027 | 0.103 | 0.011 | 0.111 | 0.012 |
| SSD | 0.084 | 0.007 | -0.039 | 0.002 | 0.035 | 0.001 |
| PDD | -0.239 | 0.057 | -0.099 | 0.010 | -0.061 | 0.004 |
| CD | -0.043 | 0.002 | -0.079 | 0.006 | -0.035 | 0.001 |
| ODD | -0.045 | 0.002 | -0.048 | 0.002 | -0.035 | 0.001 |
| AN | -0.132 | 0.017 | -0.182 | 0.033 | -0.104 | 0.011 |
| BN | -0.127 | 0.016 | -0.207 | 0.043 | -0.152 | 0.023 |
| OCD | -0.002 | 0.000 | 0.045 | 0.002 | -0.028 | 0.001 |
| SLDR | 0.260 | 0.068 | 0.140 | 0.020 | 0.074 | 0.005 |
| SLDW | 0.254 | 0.065 | 0.220 | 0.048 | 0.105 | 0.011 |
| SLDM | 0.057 | 0.003 | 0.101 | 0.010 | 0.351 | 0.123 |
| PTSD | -0.129 | 0.017 | -0.072 | 0.005 | -0.016 | 0.000 |

Value r (correlation) ≥ .15 [or r^2^ (variance ≥ .023)] significant at *p* =.05.

*Note*. GAD = Generalised Anxiety Disorder; SAD = Separation Anxiety Disorder, ADHDHI = Attention-Deficit/Hyperactivity Disorder: Hyperactive/Impulsive; ADHDI = Attention Deficit/Hyperactivity Disorder: Inattentive; ASD = Autism Spectrum Disorder; LD = Language Disorder; SSD =Speech Sound Disorder; PDD = Persistent Depressive Disorder; CD = Conduct Disorder; ODD = Oppositional Defiant Disorder; AN = Anorexia Nervosa; BN = Bulimia Nervosa; OCD = Obsessive-Compulsive Disorder; SLDR = Specific Learning Disorder: Reading; SLDW = Specific Learning Disorder: Written Expression; SLDM = Specific Learning Disorder: Mathematics; PTSD = Posttraumatic Stress Disorder; WAIT-III= Wechsler Individual Achievement Test, Third Edition; Read = WIAT-III reading; Written = WIAT-III writing; Maths = WIAT-III mathematics.
